# A Flood Damage Allowance Framework for Coastal Protection With Deep Uncertainty in Sea Level Rise

**DOI:** 10.1029/2019EF001340

**Published:** 2020-03-10

**Authors:** D. J. Rasmussen, Maya K. Buchanan, Robert E. Kopp, Michael Oppenheimer

**Affiliations:** ^1^ Woodrow Wilson School of Public and International Affairs Princeton University Princeton NJ USA; ^2^ Climate Central Princeton NJ USA; ^3^ Department of Earth and Planetary Sciences Rutgers University Piscataway NJ USA; ^4^ Institute of Earth, Ocean, and Atmospheric Sciences Rutgers University New Brunswick NJ USA; ^5^ Department of Geosciences Princeton University Princeton NJ USA

**Keywords:** sea level rise, coastal flooding, Antarctica, deep uncertainty, damage, public policy

## Abstract

Deep uncertainty describes situations when there is either ignorance or disagreement over (1) models used to describe key system processes and (2) probability distributions used to characterize the uncertainty of key variables and parameters. Future projections of Antarctic ice sheet (AIS) mass loss remain characterized by deep uncertainty. This complicates decisions on long‐lived coastal protection projects when determining what margin of safety to implement. If the chosen margin of safety does not properly account for uncertainties in sea level rise, the effectiveness of flood protection could decrease over time, potentially putting lives and properties at a greater risk. To address this issue, we develop a flood damage allowance framework for calculating the height of a flood protection strategy needed to ensure that a given level of financial risk is maintained. The damage allowance framework considers decision maker preferences such as planning horizons, protection strategies, and subjective views of AIS stability. We use Manhattan—with the population and built environment fixed in time—to illustrate how our framework could be used to calculate a range of damage allowances based on multiple plausible scenarios of AIS melt. Under high greenhouse gas emissions, we find that results are sensitive to the selection of the upper limit of AIS contributions to sea level rise. Design metrics that specify financial risk targets, such as expected flood damage, allow for the calculation of avoided flood damages (i.e., benefits) that can be combined with estimates of construction cost and then integrated into existing financial decision‐making approaches (e.g., benefit‐cost analysis).

## Key Points

- Not accounting for sea-level rise when designing ood protection could cause the originally selected margin of safety to decrease over time
- Damage allowances provide the heights of flood protection strategies needed to maintain the expected flood damage as sea levels rise
- Benefit‐cost analysis used for flood protection decision‐making should consider multiple scenarios of Antarctic ice sheet melt

## 1 Introduction

Rising sea levels increase the expected frequency of extreme sea level (ESL) events of a given height (Buchanan et al., 2017; Vitousek et al., 2017; Vousdoukas et al., 2018; Wahl et al., 2017). For example, a 0.5‐m increase in mean sea level at the Battery in lower Manhattan (New York City [NYC], USA) would increase the expected frequency of the local 100‐year ESL event from once every 100 years to about once every 20 years (Rasmussen et al., 2018). This poses a challenge to designers of coastal flood protection strategies that seek to maintain a given margin of safety over time (e.g., protection against the 100‐year flood). Without accounting for sea level rise (SLR) in the design of coastal flood protection the originally selected margin of safety could decrease, potentially leading to increased flood damages and greater numbers of people at risk. To deal with this issue, ESL hazard “allowances” have been developed (Buchanan et al., 2016; Hunter, 2012; Slangen et al., 2017). A hazard allowance is the vertical distance an asset needs to be raised in order to ensure that the expected number of ESL events is kept constant under SLR.

We note two limitations of hazard allowances. First, hazard allowances only consider the heights of physical water levels and not their damages. If risk is characterized by the probability of a hazard and its consequence (Kaplan & Garrick, 1981), then the assessment of the benefits of coastal risk reduction measures requires consideration of both the probability of an ESL event and subsequent damages. An allowance that maintains financial risk over time (e.g., the annual average loss [AAL] due to flooding) rather than a physical hazard (e.g., ESLs) directly quantifies reductions in damage and could better inform financial decision‐making (e.g., benefit‐cost analysis, BCA). Second, future projections of global mean sea level (GMSL) are characterized by “deep uncertainty” (Lempert et al., 2003). Deep uncertainty (also synonymous with Knightian uncertainty and Ellsbergian “ambiguity”) describes situations where there is either ignorance or disagreement by experts or decision makers over (1) conceptual models used to describe key system processes and (2) probability distribution functions (PDFs) used to characterize uncertainty related to key variables and parameters (Weyer, [Link]). Incomplete understanding of the physical processes that govern the behavior the Antarctic ice sheet (AIS) inhibits the characterization of a single, unambiguous PDF of future GMSL under a given emissions scenario (Bakker et al., 2017; Cozannet et al., 2017; Kopp et al., 2017; Kopp et al., 2019). Considerably different probabilistic projections can result from differing physical modeling approaches. Therefore, coastal decision‐making approaches should accommodate multiple plausible PDFs of AIS melt (e.g., Kopp et al., 2019; Slangen et al., 2017; Wong & Keller, 2017). In this study, we address these two limitations by linking ESLs to financial loss using a simple, time‐invariant damage function that is modified based on the flood protection strategy used and also employ future probabilistic projections of local SLR that accommodate multiple subjective beliefs regarding future AIS behavior.

Flood mitigation strategies can produce significant benefits to society by reducing damage to buildings and infrastructure and can potentially save lives (Aerts et al., 2014; Lincke & Hinkel, 2018; Scussolini et al., 2017). Without additional investments in adaptation measures, by the end of the century direct damages from coastal floods on the global scale could exceed one trillion U.S. dollars per year (Diaz, 2016; Hinkel et al., 2014; Jevrejeva et al., 2018), with most losses occurring in highly exposed coastal cities (Hanson et al., 2011; Hallegatte et al., 2013). In response, many of these urban areas are exploring or implementing flood protection strategies (e.g., Merrell et al., 2011; UK Environment Agency, 2012; Pirazzoli & Umgiesser, 2006; SIRR, 2013). These strategies can be categorized as accommodation, defense, advance, and retreat (Oppenheimer et al., [Link]). Accommodation reduces damage when inundation occurs (e.g., elevating structures or other flood‐proofing measures), while defense seeks to prevent inundation using structural measures such as levees and storm surge barriers. Advance creates new lands by building into the sea and retreat permanently moves assets and populations away from the coastline. The height of a protection strategy (i.e., the design height) is a key variable as it is generally consistent with the reduction in the AAL due to flooding; the greater the height, the larger the reduction (all else being equal). A poorly selected design height could lead to greater residual risks in terms of public safety and damage or overprotection and unnecessary spending.

Formal decision‐making approaches can aid in the appraisal of flood protection strategies, including design height calculations. For example, BCA is commonly used by government agencies to economically optimize design heights by balancing incremental reductions in risk with incremental investments in greater margins of safety (e.g., Fankhauser, 1995; Kanyama et al., 2019; Ramm et al., 2017; van Dantzig, 1956). Different decision approaches have different variables that can be prescribed by users (e.g., construction cost and discount rates). For BCA, the margin of safety is not a parameter that can be specified (e.g., protection against the 100‐year ESL event) but rather a variable to be solved for (e.g., by maximizing expected net present value). This approach may be insufficient if a specific margin of safety is desired. On the other hand, hazard allowance frameworks specify a margin of safety (Buchanan et al., 2016), but design heights are calculated without regard to cost.

To fill the space between these two existing decision approaches, we develop a flood “damage allowance” framework that facilitates estimation of the design heights of flood protection strategies aimed at maintaining a user‐defined level of risk (i.e., AAL) under multiple assumptions of future AIS mass loss (i.e., the damage allowance). We consider four protection options: elevation, coastal retreat, a levee, and a storm surge barrier. Advance is not included due to complexities associated with projecting property growth in new lands. If avoided damages from each protection option are considered benefits, they could be combined with the costs of implementing each approach (not quantified in this study) and then input into a BCA or cost‐effectiveness framework (e.g., Aerts et al., 2014; Scussolini et al., 2017). We use Manhattan (NYC) to illustrate framework application, but note that in this study we focus on the development of the flood damage allowance framework and give less attention to developing rigorous estimates of current and future flood damages. Users can employ the damage allowance framework with their preferred flood modeling approach or damage functions.

## 2 Framework

**Figure 1:**
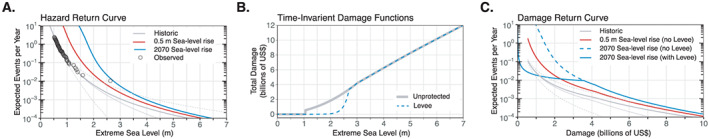
(a) Expected number of extreme sea level (ESL) events per year as a function of ESL height (meters above mean higher high water) at the Battery (Manhattan, New York City) for historical mean sea level (gray lines), 0.5 m of sea level rise (SLR; red line), and projected SLR in 2070 (blue line). Thin gray lines are the historical ESL height return curves for the 5th/50th/95th percentiles of the generalized Pareto distribution parameter uncertainty range (dotted/solid/dotted lines, respectively). Tide gauge observations (1920–2014) are plotted as open black circles. (b) Direct physical flood damage in Manhattan (billions of 2017 US$) as a function of ESL height (meters above mean higher high water) as estimated by a time‐invariant flood damage function (section SA5) that assumes a 1.0‐m‐high bulkhead around Manhattan (gray curve) and a time‐invariant flood damage function that assumes a 1.7‐m‐high levee on top of the bulkhead protecting Manhattan that includes the probability of structural failure below the top of the levee (dashed blue curve; section 2.1.2). (c) As for (a) but for the expected number of flood damage events per year (billions of 2017 US$) for historical mean sea level (gray lines), 0.5 m of SLR and no added protection (red line), projected SLR in 2070 with no added protection (dash blue line), and projected SLR in 2070 with a 1.7‐m levee that maintains the historical AAL due to flooding (solid blue line). The projected damages assume that Manhattan's distribution of buildings, people, and infrastructure remains constant in time.

The flow and sources of information used in our framework are shown in Figure A1 in the supporting information (SI). Additional details and limitations to our approach are given in the SI. First, we estimate the present‐day probability of ESLs of various heights by applying extreme value theory to a long‐term record of sea level observations (Figure 1a; section SA1). Second, increases in ESL frequency are accounted for using probabilistic, local SLR projections (section SA3) that have been adjusted by (1) subjectively weighting the likelihood of rapid AIS mass loss mechanisms and (2) specifying an upper limit to 2100 AIS contributions to GMSL (section 2.2). Third, we develop a simple, aggregate flood damage function using (1) a “bathtub” approach to model the spatial variation in floods (section SA4), (2) building stock data, and (3) observed relationships between flood depth and building type (Figure 1b; section SA5). The damage function assumes a “frozen city,” in that the population and assets remain in place over time (except when modeling retreat). Fourth, a flood protection strategy is chosen that modifies the shape of the damage function (e.g., levee; Figure 1b). Fifth, the location's AAL due to flooding is projected into the future using the damage function and the PDF of ESLs under SLR (Figure 1c). Finally, the design height of a given flood mitigation strategy is calculated such that the future AAL due to flooding under uncertain SLR equals a user‐specified level of acceptable financial risk (e.g., the current AAL).

### 2.1 A Generalizable Damage Stabilizing Model

If $f(z^{*})$ is the current annual exceedance probability (AEP) of a given ESL event with surge height $z^{*}$ (e.g., the 100‐year event), then the instantaneous hazard allowance $A(z^{*})$ that maintains the AEP under uncertain sea level change Δ can be expressed as follows:

(1) $$f(z^{*})=\int_{\Delta }f(z^{*}-\Delta +A(z^{*}))P(\Delta )\;d\Delta ,$$

where $f(z^{*}-\Delta )$ is the AEP of $z^{*}$ after including sea level change Δ whose uncertainty is given by the PDF, P(Δ). For a given AEP, the hazard allowance can be interpreted as the horizontal distance between the expected historical and future ESL return curve (Figure 1). If Δ is known, then A=Δ, but if SLR projection uncertainty is considered, the hazard allowance will always be greater than the expected SLR due to an approximately log‐linear relationship between the AEP of ESLs and water height (Buchanan et al., 2016).

We extend the hazard allowance concept to damage events by employing a simple, time‐invariant damage function that describes the relationship between ESLs and direct physical damages. The damage allowance is the design height of a flood mitigation strategy needed to maintain the current frequency of damage events under uncertain SLR (i.e., the AAL due to flooding, calculated by integrating under the damage return curve). To offset additional damages due to SLR, we create a “protected” damage function using idealized representations of how the flood damage reduction strategies of elevation (section 2.1.1), a levee or storm surge barrier (section 2.1.2), and coastal retreat (section 2.1.3) could impact the relationship between ESLs and damage in the “unprotected” damage function. In order to stabilize the AAL resulting from uncertain SLR, the current AAL must equal the projected AAL that includes both an arbitrary sea level change ( Δ) plus an adjustment to offset the increase in damages resulting from SLR. This “conservation of damage” can be mathematically represented by the following:

(2) $$\int_{A_{min}}^{\infty }\int_{\Delta }D^{*}(z)f(z-\Delta )P(\Delta )\;d\Delta \;dz=\int_{A_{min}}^{\infty }D(z)f(z)\;dz,$$

where z is the ESL height, $D^{*}(z)$ is a protected damage function that includes adjustments to mitigate additional flood damage resulting from SLR depending on the adaptation strategy taken (elevation, levee, storm surge barrier, or coastal retreat; sections 2.1.1, 2.1.2, 2.1.3), D(z) is the unprotected damage function (section SA5), and $A_{min}$ is the current protection height. While all allowances traditionally aim to maintain the current level of risk (i.e., the historical AAL), we note that this could be replaced by any user‐specified level of risk. In the SI, we show how equation (2) can return equation (1) (section SA7).

#### 2.1.1 Damage Reduction Strategy: Elevation

**Figure 2:**
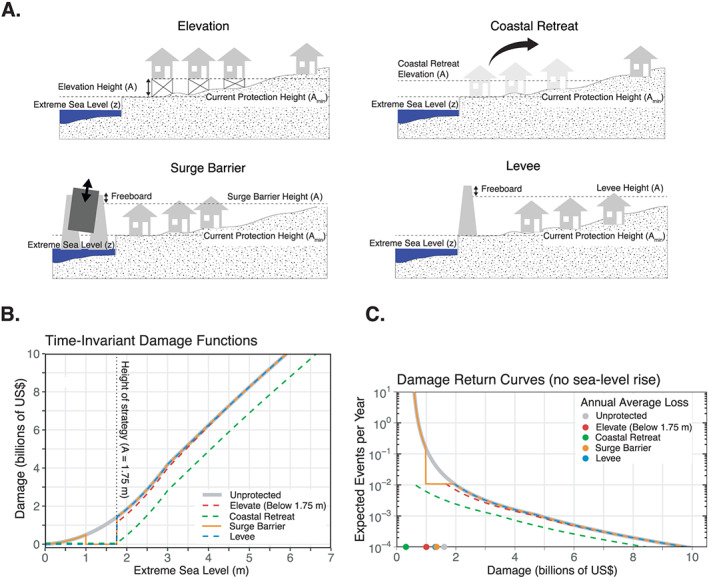
(a) Schematics illustrating each flood protection strategy for an arbitrary protection height ( A). (b) Time‐invariant damage functions for Manhattan that relate extreme sea level (ESL; meters) to total direct damage due to flooding (billions of US$; section SA5). The thick gray line is the unprotected damage function that assumes no existing bulkhead around Manhattan ( $A_{min}$ = 0); the dashed red line is the damage function after elevating all structures 1.75 m below 1.75 m in elevation; the dashed green line is the damage function for coastal retreat of all structures below 1.75 m of elevation; the solid orange line is the damage function for a storm surge barrier with a protection height of 1.75 m with gates that close when the ESL is >1.0 m and has zero probability of structural failure and no freeboard; the dashed blue line is the damage function for a levee with a protection height of 1.75 m that has zero probability of structural failure and no freeboard. (c) Damage event return curves under no sea level rise showing the expected number of flood damage events per year (billions of 2017 US$) with no protection strategy (thick gray curve) and under the flood protection strategies of elevation (dashed red curve), coastal retreat (dashed green curve), a storm surge barrier (solid orange curve), and a levee (dashed blue curve). All cases assume that Manhattan's distribution of buildings, people, and infrastructure remains constant in time. For illustrative purposes, all cases assume no bulkhead around Manhattan ( $A_{min}$ = 0), and the storm surge barrier and levee strategies assume no possibility of structural failure and no freeboard. The discontinuity for the storm surge barrier (solid orange curve) occurs due to protection being limited to a range of ESLs (here 1.0 to 1.75 m). The AAL under each protection strategy is plotted on the x axis with a filled circle.

Accommodation strategies, such as elevating buildings on columns, reduce the vulnerability to populations and the built environment. Elevation allows for floods to occur below the design height without incurring significant damage to the structure. We model an elevation strategy that raises all structures by the vertical height A at or below the land elevation A (Figure 2a). Our approach is also consistent with wet‐proofing basements and building new floors on top of the existing structure. To estimate the height A that structures need to be elevated to maintain the current AAL under uncertain SLR, $D^{*}(z)$ is substituted in equation (2) with the following:

(3) $$D_{e}^{*}(z,A)=\underset{\text{Damage to elevated structures}}{\underbrace{\phi (z-A)\int_{e_{min}}^{A}p(e)\;de}}+\underset{\text{Damage to nonelevated structures}}{\underbrace{\int_{A}^{z}p(e)\cdot \phi (z-e)\;de,}}$$

where z is the ESL height, A is the damage allowance (above the current protection level, $A_{min}$), p(e) is the amount of property at elevation e, and ϕ(z−e) is an inundation depth‐damage function for NYC that relates the flood height ( z−e) to damage as a fraction of the total property value (see section SA6). Equation (3) is plotted in Figure 2b assuming A = 1.75 m. Note that no flood damage occurs to structures when z<A, but when z≥A, $D_{e}^{*}(z,A)$ begins to converge to the unprotected damage function D(z). A method for elevating all structures within a damage function is also provided (section SA8).

#### 2.1.2 Damage Reduction Strategies: Levee and Storm Surge Barrier

Flood defenses physically block the inland advance of ESLs and include both hard (e.g., levees and storm surge barriers) and soft (e.g., beach‐dune systems) approaches. Levees are stationary embankments strategically used to prevent areas from flooding. While they may not require private actors to undertake action (e.g., purchasing insurance, elevation, or retreat), levees have the disadvantage of maximal loss occurrence by either being overtopped (e.g., by experiencing ESLs outside of the designed margin of safety) or by suffering structural failure (breaching). We model the probability of levee failure ( $p_{f}$) conditional on the ESL z (i.e., the structural load), the targeted design height A, the failure rate for the water load at the design height ( $tol_{A}$; assumed to be 0.10, which is the minimum threshold for the probability of a loading breach for Dutch flood defenses; TAW, 1998), and the height of the freeboard $F_{b}$ (i.e., arbitrary additional protection above A): $p_{f}=P(fail\;|\;z,A,tol_{A},F_{b})$. According to Wolff (2008), for a well‐designed levee, the probability of failure below the design height should be “unlikely” and then increase rapidly until it reaches unity at the targeted design height plus the specified freeboard. We model $p_{f}$ using an exponential relationship that is a function of z, $p_{f}=a\cdot \exp(b\cdot z)$, where $b=\ln(1/tol_{A})/F_{b}$ and $a=\exp(-b\cdot A+\ln(tol_{A}))$ (Figure SA7). For levees, $D^{*}$ is substituted in equation (2) with

(4) $$D_{l}^{*}(z)=p_{f}(z,A)D(z).$$

In other words, total damage scales with the probability of levee failure until overtopping occurs (Figures 2b and SA7). The base of the levee is assumed to rest on top of the existing flood protection ( $A_{min}$; Figure 2).

Storm surge barriers are gates placed within bodies of water (usually tidally influenced rivers or estuaries) that remain open to allow for tidal flushing and maritime navigation but close during forecasted ESLs (e.g., coastal storms). Storm surge barriers are often placed within levee systems (Mooyaart & Jonkman, 2017). We model storm surge barriers similarly to levees but include a parameter that governs gate closure. Due to increased chances of mechanical failure, environmental impacts, and shipping disruption, the frequency of storm surge barrier gate closure is often restricted (Sustainable Solutions Lab, 2018). This prevents storm surge barriers from protecting against more frequent events that can cause minor flooding, such as extreme tides (Sweet et al., 2016). Some of the largest storm surge barriers are designed to close approximately once every 10 years, such as the Maeslant Barrier (Netherlands), while smaller‐ to medium‐sized barriers, such as the Thames Barrier (United Kingdom), have historically been designed to close no more than two to three times per year (Sustainable Solutions Lab, 2018). Figure 1a shows the modified damage function for a surge barrier that closes when z ≥ 1.0 m. For storm surge barriers, $D^{*}$ is substituted in equation (2) with

(5) $$D_{b}^{*}(z)=H_{b}[z]D(z),$$

where $H_{b}$ is

(6) $$H_{b}[z]=\left\{\begin{array}{l} \begin{array}{ccccc} & p_{f}(z,A), & & z\geq z_{close}, & \\ & 1, & & z<z_{close}. \end{array} \end{array}\right.$$

#### 2.1.3 Damage Reduction Strategy: Coastal Retreat

Coastal retreat can be described as the reduction in exposure to ESLs through the removal of building stock and populations below a specified elevation A in order to reduce expected flood damages. Such action could occur through voluntary migration, forced displacement, or planned relocation. Retreat is the only strategy that completely eliminates residual risks if there is perfect retreat compliance (i.e., 100% of population moves). Mathematically, coastal retreat modifies the unprotected damage function D(z) by subtracting out damages below A that would have occurred had they not retreated (Figure 2b). This implies that building stock below A is removed from the damage function. This also means that building stock and populations on the risk map below A are also removed. We do not allow for populations to move to higher elevations within the study area; they are assumed to move to a different region. The protected damage function is

(7) $$D_{r}^{*}(z)=D(z)H_{r}[z],$$

where $H_{r}$ is

(8) $$H_{r}[z]=\left\{\begin{array}{l} \begin{array}{ccccc} & 1-\alpha , & & z<A, & \\ & 1-\alpha \frac{D(A)}{D(z)}, & & z>A, \end{array} \end{array}\right.$$

and α is the fraction of assets and populations below A that have retreated (i.e., the retreat compliance; α∈[0,1]). Perfect compliance should not be expected, especially if retreat is voluntary. Some risk targets may not be achievable using coastal retreat if compliance is not high enough. As retreat compliance decreases, $D_{r}^{*}(z)$ approaches the unprotected damage function (Figure SA8).

### 2.2 Uncertainty in AIS Collapse

Beyond midcentury, the dynamic response of the AIS to warming is a key uncertainty in protecting future sea levels. Several different plausible estimates of continental‐scale AIS melt exist (e.g., Bamber & Aspinall, 2013; Edwards et al., 2019; Deconto & Pollard, 2016; Golledge et al., 2019; Levermann et al., 2014; Little et al., 2013; Little et al., 2013; Ritz et al., 2015), but there is currently not an agreed upon full range of outcomes and likelihoods necessary for risk assessment using a single SLR PDF. Recent modeling of ice sheet behavior, discussed in detail in Oppenheimer et al. ([Link]) and Kopp et al. (2019), demonstrates divergent PDFs and likelihoods of a partial collapse of the AIS from different modeling approaches. An implication of this ambiguity is that the PDF of SLR in the second half of the century remains strongly dependent upon subjective assessment of potential AIS contributions. Employing multiple PDFs based on such assumptions is one method to characterize this deep uncertainty (e.g., Kopp et al., 2017; Rohmer et al., 2019Jul, 2019Jul; Slangen et al., 2017; Sriver et al., 2018; Wong & Keller, 2017; Wong, Bakker, et al., 2017).

Imprecise probability methods can be used in cases where multiple PDFs cannot be reduced to a single distribution. For example, a probability box (or “p‐box”) can be used to express SLR incertitude by constraining plausible cumulative distribution functions of SLR within a defined space (Baudrit et al., 2007; Cozannet et al., 2017). If it is assumed that the upper (i.e., right edge) and lower (i.e., left edge) limits of the p‐box contain the unknown distribution, then the true probability of exceeding a given amount of SLR lies within the bounds of the cumulative distribution functions. A p‐box is shown in Figure 5a for 2100 local SLR at the Battery tide gauge in lower Manhattan. Here, we limit the upper p‐box boundary with local probabilistic SLR projections from Kopp et al. (2017), which employ the fast ice loss AIS projections from Deconto and Pollard (2016), and the lower p‐box boundary with SLR projections from Kopp et al. (2014), which include more sluggish AIS mass loss based on a combination of the Intergovernmental Panel on Climate Change's Fifth Assessment Report and expert elicitation of total ice sheet mass loss from Bamber and Aspinall (2013). These SLR projections were chosen for illustrative purposes only. For instance, a more credible approach could be to employ the more recent projections from Bamber et al. (2019) that include new expert elicitation of AIS behavior. End‐of‐century SLR projections from Bamber et al. (2019) under Representative Concentration Pathway 8.5 (RCP8.5) largely fall in between those from Kopp et al., 2014 (2014, 2017) but have a higher probability of SLR ≥ 3 m (Figure SB6).

Subjective beliefs regarding future AIS behavior are used to generate an “effective” SLR distribution within the p‐box (section SA11). The “effective” distribution is generated by (1) selecting an upper limit to 2100 AIS contributions ( ${\text{AIS}}_{max}$) and (2) averaging the upper and lower bounds of the p‐box using a weight that reflects the subjective likelihood of AIS collapse initiation before 2100 ( $\beta_{c}\in [0,1]$). Larger values of $\beta_{c}$ imply a higher likelihood of AIS collapse initiation before 2100, while lower values of $\beta_{c}$ imply a lower likelihood. We consider ${\text{AIS}}_{max}$ scenarios of 1.75, 1.5, 1.0, 0.5, and 0.25 m (relative to 2000). More details regarding the SLR projections and the p‐box construction are given in the SI (sections SA3 and SA11, respectively). Similar approaches that weight worst‐case outcomes for SLR and other climate variables have been used for decision‐making under deep uncertainty (Buchanan et al., 2016; McInerney et al., 2012).

### 2.3 Damage Allowance Framework Decision Sequence

**Figure 3:**
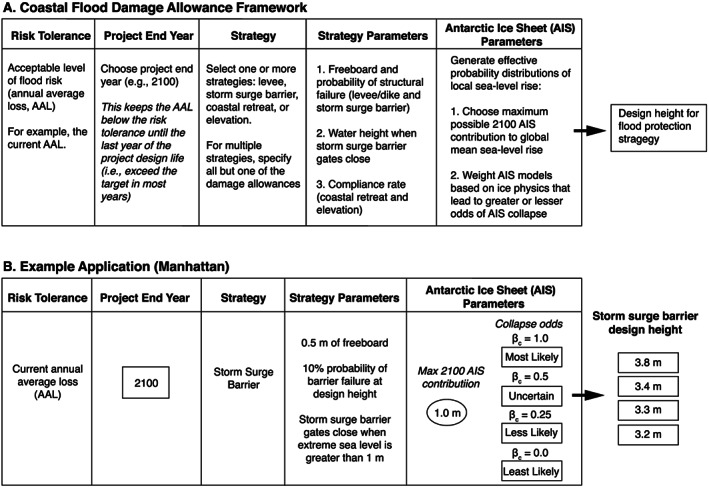
(a) Flowchart illustrating how to apply the coastal flood damage allowance framework. (b) An example application for Manhattan seeking to maintain the current annual average loss from flood damages using a storm surge barrier.

The flow of the damage allowance framework is presented in Figure 3a. First, an acceptable AAL risk target is selected. This could be the current AAL to maintain the current level of flood risk (i.e., the traditional allowance definition) or any value greater than zero. Second, both the time frame and the approach for meeting the risk target are chosen. We only consider the instantaneous allowance (i.e., risk is kept below the target through the duration of the project and is met in the final year); however, this could alternatively be a period of years over which the mean AAL equals the risk target (earlier years are below the risk target and later years are above it; i.e., the average annual design life allowance, section SA9). Third, a flood damage mitigation strategy is selected. While combined protection strategies are possible (e.g., retreat and a levee; section SA10), in this paper we illustrate damage allowances using only a single option. Finally, two parameters that govern AIS contributions to SLR are subjectively chosen: ${\text{AIS}}_{max}$ and $\beta_{c}$ (section 2.2).

## 3 Illustration of Damage Allowance Framework: Manhattan, NYC

**Figure 4:**
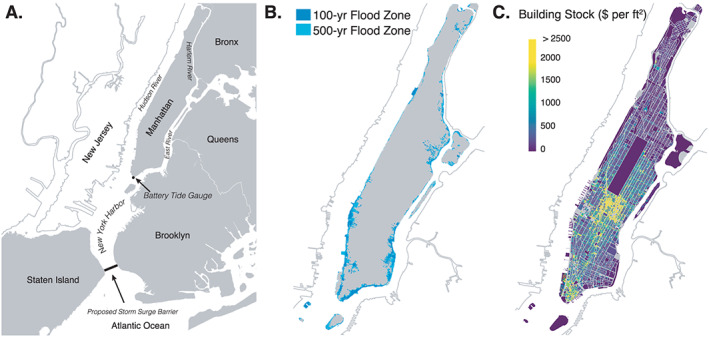
(a) A map showing Manhattan and the surrounding boroughs, the location of the Battery tide gauge in lower Manhattan, and the location of a proposed storm surge barrier at the entrance to New York Harbor. (b) Estimated 100‐ (blue) and 500‐year (cyan) flood zones in Manhattan generated using the “bathtub” approach that estimates spatial flood extents using (1) extreme sea level return periods from the Battery tide gauge and (2) a 0.3‐m horizontal resolution light detection and ranging (LiDAR)‐derived digital elevation model for the City of New York (https://data.cityofnewyork.us/City-Government/1-foot-Digital-Elevation-Model-DEM-/dpc8-z3jc) (c) The spatial distribution of tax assessed building stock value in Manhattan (excludes land value). Building stock value is given as tax assessed building value per square foot of building lot area (2017 US$ per square feet). Data are from the NYC Department of City Planning (NYC Planning, 2018).

We use Manhattan to illustrate our damage allowance framework. Manhattan is an island, surrounded by the Hudson, East, and Harlem Rivers, and is the economic and administrative center of NYC (Figure 4a). NYC ranks among the top urban areas in the world in terms of current assets and population exposed to coastal floods (Hallegatte et al., 2013; Hanson et al., 2011). In Manhattan alone, more than $50 billion (All monetary values in this paper are given in 2017 U.S. dollars [US$].) of property lies within the 100‐year flood plain (Figures 4b and 4c; NYC Comptroller, 2014). The AAL due to flooding in Manhattan is expected to increase with SLR (Gornitz et al., 2019; Orton et al., 2019). Using our flood damage model, we estimate that the current AAL could increase by more than a factor of 10 by 2070, from $0.1 billion/year to ∼$1.6 billion/year (Figure 1c). As such, NYC is exploring multiple public flood protection strategies (USACE, 2016; 2019; SIRR, 2013; NYC, 2019; NYC, 2019). A direct comparison of the current AAL with other studies is difficult due to differences in geographic scope and flood protection assumptions. Nonetheless, Aerts et al. (2014) and Houser et al. (2015) found an AAL of $0.18 and $0.53 billion/year for all of NYC, respectively, and Hallegatte et al. (2013) found an AAL of $0.79 billion/year for the entire NYC‐Newark, New Jersey, region. We note that due to the simplifications made in estimating flood risk (sections SA4–SA6), the options presented in this study are not intended to be specific recommendations for Manhattan. We also do not fully address the feasibility of implementation (e.g., financial or technical). For example, it may be impractical to elevate high‐rise structures (However, roughly 88% of all buildings in Manhattan sited at ≤8 m in elevation [relative to mean higher high water, MHHW] have less than six floors above ground level; NYC Planning, 2018. These buildings tend to be older, and their relation to the total fraction of damage is unclear.) or flood‐proof all exposed buildings. In the case of coastal retreat, we assume that populations associated with removed property move to either a different borough of NYC or elsewhere. More detailed assessments could be used to further investigate the feasibility of preferred courses of action. Manhattan is used because there is (1) a tide gauge with a long record from which to construct an ESL distribution (https://tidesandcurrents.noaa.gov/stationhome.html?id=8518750), (2) a fairly uniform tidal range around Manhattan (Orton et al., 2019), (3) freely available building stock data (NYC Planning, 2018), and (4) a relatively uniform level of existing “flood protection,” a 1.0‐m bulkhead (relative to MHHW (This is approximately the height with which water begins to flow over the bulkhead at the Battery in lower Manhattan (retrieved from https://water.weather.gov/ahps; July, 2019). This is also a simplification, as the bulkhead height varies around the island of Manhattan (1.25 to 1.75 m above mean sea level or 0.5 to 1 m above MHHW); Colle et al., 2008).)

We consider changes to the flood damage function and return curve if Manhattan implemented a levee, storm surge barrier, elevation, or coastal retreat (Figures 2b and 2c, respectively). For illustrative purposes, the 1.0‐m bulkhead around Manhattan is not considered here. A hypothetical 1.75‐m levee would prevent all flood damage events < $2 billion and would decrease the current AAL from $1.6 billion/year to $1.2 billion/year (assuming no bulkhead). The effect of a 1.75‐m storm surge barrier is similar to the levee but reduces the current AAL slightly less because ESLs < 1 m are allowed to occur when barrier gates are open (decreases current AAL from $1.6 billion/year to $1.3 billion/year). Raising all existing structures with first‐floor elevations of 1.75 m or less to 1.75 m reduces the AAL similar to that of the levee and surge barrier, from $1.6 to $1.0 billion/year. While overtopping does not occur with the elevation strategy, we note that the failure of elevated structures is possible, but not considered in this study. Of all options explored, coastal retreat reduces the current AAL the most. If all existing structures with first‐floor elevations of 1.75 m or less retreat, the AAL is reduced from $1.6 to $0.3 billion/year. Coastal retreat not only eliminates damage to building stock ≤ 1.75 m but also eliminates the possibility of damage to these structures for ESLs >1.75 m because they are removed from the damage function. For both the levee and storm surge barrier, we note that levee and barrier failure is not depicted in Figure 2b or Figure 2c but is considered in the damage allowance calculation (section 2.1.2).

An application of the damage allowance framework for Manhattan is shown in Figure 3b. In this example, a government planner chooses to maintain Manhattan's current AAL due to flooding through 2100 using a storm surge barrier placed across the tidal straight between Staten Island and Brooklyn (Figure 4a). We assume that the surge barrier would be similar in design to that of the Maeslant Barrier (Netherlands), designed to close no more than once every 10 years on average under current mean sea levels. This corresponds to an ESL closure threshold ( $z_{close}$) of roughly 1.0 m above MHHW, as estimated from Figure 1a. We assume the barrier has a 10% probability of failure at the design height, 0.5 m of freeboard, and that the 2100 AIS mass loss contributions to GMSL is ≤ 1.0 m (relative to 2000) and collapse initiation of the AIS is “less likely” ( $\beta_{c}$ = 0.25). According to the framework, the storm surge barrier should be built to a height of 3.3 m above MHHW. This increases by 0.5 m if the AIS collapse odds are instead believed to be “most likely” ( $\beta_{c}$ = 1.0).

## 4 Results

### 4.1 Characterizing Deep Uncertainty: SLR and ESL Return Periods

**Figure 5:**
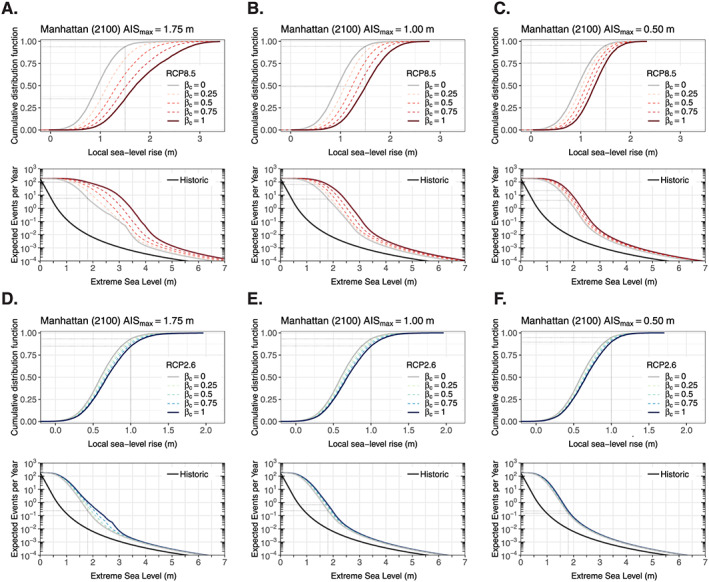
(a) Top row: probability boxes (“p‐boxes”; solid lines) for 2100 local sea level rise (SLR) in Manhattan (located at the Battery tide gauge) under the Representative Concentration Pathway (RCP) 8.5 climate forcing scenario. Effective cumulative distribution functions (CDFs) of local SLR (dashed lines) are generated within each p‐box by averaging the p‐box edges using weights ( $\beta_{c}\in$ [0,1]) that reflect a user's belief of Antarctic ice sheet (AIS) collapse initiation within the 21st century (higher values reflect higher likelihood of collapse) and by constraining the maximum possible 2100 AIS melt (AIS ${}_{max}$, relative to 2000; here, 1.75 m; section 2.2). The black dotted lines highlight the probability of exceeding 1.5 or 1.0 m of local SLR (1 −CDF) under different assumptions of AIS collapse initiation (i.e., values of $\beta_{c}$). Bottom row: extreme sea level (ESL) return curves for Manhattan showing the relationship between the expected number of ESLs per year and ESL height (meters above mean higher high water) for (1) historical sea levels (black curve) and (2) year 2100 (RCP8.5) for different values of $\beta_{c}$. All curves incorporate generalized Pareto distribution parameter uncertainty (section SA1), and the future return curves additionally incorporate local SLR projection uncertainty by integrating across the entire local SLR probability distribution. The black dotted lines highlight the annual expected number of historically experienced 100‐year ESL events under different values of $\beta_{c}$. (b) As for (a) but for AIS ${}_{max}$ = 1.0 m. (c) As for (a) but for AIS ${}_{max}$ = 0.5 m. (d) As for (a) but for RCP2.6. (e) As for (a) but for RCP2.6 and AIS ${}_{max}$ = 1.0 m. (f) As for (a) but for RCP2.6 and AIS ${}_{max}$ = 0.5 m.

In Figure 5, we illustrate deep uncertainty associated with 2100 local SLR and ESLs in Manhattan under multiple values of ${\text{AIS}}_{max}$ for both RCP8.5 and RCP2.6 (high and low climate forcing scenarios, respectively). Deep uncertainty is explored within each p‐box under multiple assumptions regarding the likelihood and speed of collapse of the AIS ( $\beta_{c}\in$ [0,1]). Under RCP8.5 and ${\text{AIS}}_{max}$ = 1.75 m, the probability of local SLR ≥ 1.5 m is 6% under the least likely AIS collapse assumption ( $\beta_{c}$ = 0) but is 65% under the most likely AIS collapse assumption ( $\beta_{c}$ = 1; Figure 5a). The spread between these probabilities reflects the level of deep uncertainty. The sensitivity of SLR in 2100 to $\beta_{c}$ decreases as ${\text{AIS}}_{max}$ decreases. For instance, in the case of ${\text{AIS}}_{max}$ = 1.0 m, the probability of SLR ≥ 1.5 m is 6% ( $\beta_{c}$ = 0) and 51% ( $\beta_{c}$ = 1), while in the case of ${\text{AIS}}_{max}$ = 0.5 m, the probability of SLR ≥ 1.5 m is 5% ( $\beta_{c}$ = 0) and 24% ( $\beta_{c}$ = 1; Figures 5b and 5c, respectively). Deep uncertainty for the AIS is larger under RCP8.5 than RCP2.6. Under RCP2.6, assumptions regarding future AIS behavior do not significantly impact projections. For instance, for ${\text{AIS}}_{max}$ = 1.75 m, the probability of SLR ≥ 1.0 m is 7% under $\beta_{c}$ = 0 but is 14% under $\beta_{c}$ = 1 (Figure 5d). For ${\text{AIS}}_{max}$ = 0.5 m, these probabilities reduce slightly to 5% and 13%, respectively (Figure 5f). Additional p‐boxes are given in the SI for 2050 and 2070 and ${\text{AIS}}_{max}$ = 1.5 and 0.25 m (Figures SB1 to SB5).

Deep uncertainty associated with the AIS is also reflected in the projected increase in the expected number of ESL events in 2100. For instance, under RCP8.5 and ${\text{AIS}}_{max}$ = 1.75 m, the number of present‐day 100‐year ESL events in Manhattan is expected to increase from 0.01 per year, on average, to between roughly 6 per year ( $\beta_{c}$ = 0) and 100 per year ( $\beta_{c}$ = 1), on average (Figure 5a). Reducing ${\text{AIS}}_{max}$ decreases the spread of the ESL return curves under different values of $\beta_{c}$. For instance, under RCP8.5 ( ${\text{AIS}}_{max}$ = 0.5 m), the expected number of historical 100‐year ESL events is roughly 4 per year ( $\beta_{c}$ = 0) and 20 per year ( $\beta_{c}$ = 1), on average (Figure 5c). Under RCP2.6, both the absolute expected number of ESL events and the spread of the ESL return curves decreases. For instance, assuming ${\text{AIS}}_{max}$ = 1.75 m, the projected number of present‐day 100‐year ESL events is roughly between 0.2 per year ( $\beta_{c}$ = 0) and 1 per year ( $\beta_{c}$ = 1), on average (Figure 5d), but for ${\text{AIS}}_{max}$ = 1.0 m the projected number of 100‐year ESL events is approximately between 0.2 per year ( $\beta_{c}$ = 0) and 0.7 per year ( $\beta_{c}$ = 1), on average (Figure 5e). For ${\text{AIS}}_{max}$ = 0.5 m, there is little to no difference in the expected number of 100‐year flood events based on the perceived likelihood of AIS collapse; the number of expected 100‐year flood events for both $\beta_{c}$ = 0 and $\beta_{c}$ = 1 is roughly 0.2 per year, on average (Figure 5f). Additional ESL return curves are given in the SI (Figures SB1 to SB5).

### 4.2 Flood Damage Allowances: Surge Barrier and Coastal Retreat

Hazard allowances only consider the physical heights of water and do not assure that the number of damage events will simultaneously be held constant over time. ESL return curves for Manhattan are shown in Figure 1a for both Δ = 0.5 m and the entire PDF of projected 2070 SLR ( $P{\left(\Delta \right)}_{2070}$). Here, the hazard allowance A is 0.5 m for Δ = 0.5 m, but A = 0.86 m when considering $P{\left(\Delta \right)}_{2070}$. However, depending on local flood protection, an ESL event may or may not lead to damage. For example, a 1.0‐m bulkhead around Manhattan prevents ESLs ≤1 m from causing damages <∼$0.5 billion (Figure 1b). Adding a levee on top of the 1.0‐m bulkhead reduces losses for some ESL heights (Figure 1b). Under 2070 SLR, this levee needs to be built to 1.70 m above the bulkhead to maintain the current AAL (i.e., the damage allowance; Figure 1c), nearly 2 times greater than the hazard allowance (0.86 m) and more than twice as large as the expected 2070 SLR (0.80 m; Table SB1).

**Figure 6:**
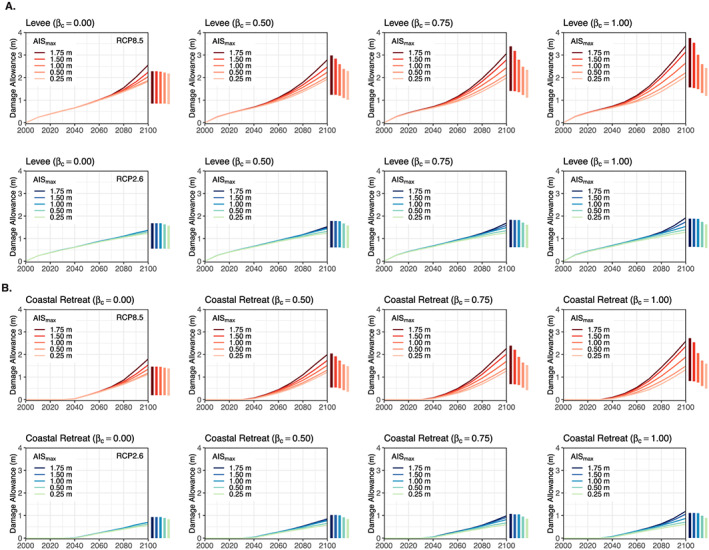
(a) Top row: Levee damage allowances (meters above the current protection height) over time (2000–2100) for protecting Manhattan under different maximum 2100 Antarctic ice sheet (AIS) contribution thresholds (AIS ${}_{max}$, relative to 2000), different subjectively perceived likelihoods of AIS collapse ( $\beta_{c}$; 0 being *most unlikely* and 1 being *most likely*), and for the Representative Concentration Pathway (RCP) 8.5 climate forcing scenario. The colored bars in the margins of each plot show the 2100 damage allowances using only the 5th/95th percentile local sea level rise projections. The levee allowances include 0.5 m of freeboard and have a 10% probability of failure at the design height. Bottom row: As for top row but for RCP2.6. (b) As for (a) but for coastal retreat (assuming perfect retreat compliance).

Damage allowances for a levee and coastal retreat that maintain the current AAL for Manhattan under different assumptions of AIS behavior are given in Figure 6 (RCP8.5 and RCP2.6). We assume 0.5 m of freeboard for the levee and a 10% failure rate (see section 2.1.2). Both damage allowances are relative to a 1‐m bulkhead above MHHW ( $A_{min}$ = 1 m). As sea levels rise, the damage allowances increase. The allowances for the levee increase faster than coastal retreat because as the levee height increases, more property becomes subjected to potential overtopping or breaching. This added risk must be compensated by an increase in levee height. The damage allowances for the levee and coastal retreat also increase at faster rate compared to local SLR (Tables SB1–SB3). For instance, the mean SLR for 2050, 2070, and 2100 is 0.4, 0.8, and 1.8 m, respectively, while the corresponding levee damage allowances are 0.9, 1.6, and 3.4 m, respectively (RCP8.5; ${\text{AIS}}_{max}$ = 1.75 m and $\beta_{c}$ = 0). For both RCPs, the assumptions regarding AIS behavior make little or no difference in the levee damage allowances before midcentury. However, after midcentury under RCP8.5, levee damage allowances may differ by up to 1.4 m, depending on ${\text{AIS}}_{max}$ and $\beta_{c}$. Under RCP2.6, differences only occur after 2080 for $\beta_{c}>$ 0.75 and are ≤ 0.6 m (Figure 6a and Table SB2). The sensitivity of allowances based on assumptions of AIS behavior is consistent with previous findings (Slangen et al., 2017). Results for a storm surge barrier and elevation are given in the SI (Figures SB7 and SB8; Tables SB4 and SB5).

### 4.3 Result Sensitivity to ESL Rise Samples

Both ESL frequencies and damage allowances are sensitive to the selection of ${\text{AIS}}_{max}$, which effectively limits the upper tail of the SLR PDF. The smaller the value of ${\text{AIS}}_{max}$, the more low probability, extreme SLR samples excluded. This is important because extreme SLR samples can strongly influence results. For example, in the case of ESL frequency, the highest samples in the SLR PDF can cause the ESL event frequency to saturate at 182.6 per year (e.g., the $\beta_{c}$ = 0 return curve in Figure 5a and the $\beta_{c}$ = 1 return curve in Figure 5d). This saturation subsequently increases the expected number of ESL events and visually appears as “kinks” in the return curves. Both the positioning and the presence of the kinks are sensitive to the choice of ${\text{AIS}}_{max}$. The kinks disappear for ${\text{AIS}}_{max}\leq$ 1.0 m (Figures 5b, 5c, 5e, and 5f). Extreme SLR samples can also increase damage allowances beyond that which might result when considering only the 95th percentile of SLR. To illustrate, we calculate damage allowances that only consider the 5th/95th percentiles 2100 SLR projections, rather than the entire SLR PDF (shown in the margins of each panel in Figure 6). For ${\text{AIS}}_{max}$ = 1.75 m and $\beta_{c}$ = 0 (RCP8.5), the damage allowances that consider only the 95th percentile SLR are lower than those that consider the entire SLR PDF. If the SLR PDF in consideration is long tailed, then illustrating allowance uncertainty based on the 5th/95th SLR percentiles may be more practical for some design applications. On the other hand, not fully considering the upper tail could underrepresent risk. Users of existing ESL and allowance frameworks should take note of the sensitivity to the truncation of the SLR PDF (e.g., Buchanan et al., 2016, 2017; Rasmussen et al., 2018).

## 5 Discussion

While simple models like flood damage allowances may make too many approximations for readily implementable final project designs, they are well suited for early planning phases when the focus is on exploring coastal protection strategies worth examining in greater detail with more complex models. Rather than being viewed as substitutes, reduced‐form models can complement their more complex peers. For example, while more complex integrated assessment frameworks may better simulate reality, they demand high computational costs, which places limits on the number of flood protection strategies that can be investigated simultaneously (e.g., Fischbach et al., 2017). Simple models may be more useful in cases where the appraisal of several project designs is needed, such as robust strategy identification (e.g., Lempert et al., 2003; Sriver et al., 2018). Additionally, exploring interactions between multiple variables with complex models can make it challenging to understand how different flood protection strategies and SLR assumptions impact benefits from proposed solutions.

Despite the mentioned advantages, there are multiple caveats associated with flood damage allowances. First, the effectiveness of flood protection is dependent on both the changing hazard (SLR, ground subsidence rates, coastal storm frequency, and severity) and changes in the consequence (e.g., what is behind the levee and how vulnerable is it to flood damage). In this study, SLR is the only variable that evolves over time. Design heights needed to meet risk tolerance targets could be higher or lower depending on these and other variables. For instance, it has been observed that well‐designed flood damage reduction strategies can lead to increased development in protected areas due to a greater sense of perceived safety (the “levee effect”; Di Baldassarre et al., 2018; White, 1945). This can increase residual flood risk over time. Additionally, from an aesthetic perspective, elevation, levee and surge barrier construction, and retreating from the floodplain could all impact building amenity value. These impacts could be important but are not considered. Second, allowances assume risk tolerance remains constant over the lifetime of the investment, during which one may wish to increase the margin of safety. Third, levees and storm surge barriers can produce storm surge funneling effects that further elevate the water surface. This, as well as the added impact of waves, is not considered and could increase the damage allowance. Fourth, damages and loss of lands from permanent inundation or coastal erosion are not accounted for. This could have a significant impact on the effectiveness of a specific flood protection strategy. In the case of a storm surge barrier, after sea level has risen above the gate closure threshold, the barrier may need to remain closed to be effective. Finally, if the damage allowances are used to produce benefits for a BCA or cost‐effectiveness framework, limitations associated with those methods also apply (e.g., Arrow et al., 1996). This includes potentially giving less weight to lower income groups. Calculations that are instead based on human population exposure could give more consideration to these demographics.

So‐called flexible/adaptive decision approaches can address challenges associated with deep uncertainty. Flexible/adaptive decision approaches commit to short‐term actions in response to new information (e.g., Haasnoot et al., 2013; Walker et al., 2013; Wise et al., 2014). They have the advantage of being less dependent on accurate projections of the future (e.g., flexible levee design allowing for heightening over time as risk tolerances change or as new information is learned about hard‐to‐predict variables). Despite this key advantage, there are multiple political reasons that a multiple‐priors approach (like flood damage allowances) might be pursued over a flexible/adaptive framework.

Efforts to use flexible/adaptive approaches could be complicated by existing laws and government agency protocols that promote BCA and cost‐effectiveness analysis. For example, the U.S. Army Corps of Engineers, the principal agency tasked with designing and implementing coastal flood risk management projects in the United States, uses future projections of SLR in their BCA to assess and select coastal protection strategies that best “contribute to national economic development consistent with protecting the Nation's environment” (Public Law, 1936; U.S. Water Resources Council, 1983; USACE, 2019). Second, financing for disaster preparedness projects is usually only available following major disasters (NRC, 2014), in part due to motivations of elected officials (Healy & Malhotra, 2009). Current flood protection revenue streams are inadequate for supporting either new construction or regular upgrades that may occur with a flexible/adaptive approach (Knopman et al., 2017; Sustainable Solutions Lab, 2018). This financing arrangement may reinforce the use of prediction‐first approaches that force a decision to be made now with no planned opportunity to revisit the course of action in the future. Third, flexible/adaptive approaches may be more expensive compared to designing once (Fankhauser et al., 1999; Haasnoot et al., 2019), and they may involve more political overhead to change existing governance structures away from appraisals based on BCA (e.g., Kanyama et al., 2019; Ramm et al., 2017). This could delay making a decision regarding what flood protection strategy to pursue during which additional flood damages may occur. Development times for building flood protection are already long. For example, experience with storm surge barriers has shown that it can take decades to design and build a multibillion dollar project (Morang, 2016; Sustainable Solutions Lab, 2018).

## 6 Conclusions

A number of formal decision‐making frameworks exist for designing strategies that mitigate flood damages (Walker et al., 2013). These are generally driven by economic objectives, such as choosing courses of action where monetized discounted benefits exceed discounted costs (i.e., BCA). We introduce flood damage allowances as a new approach for determining the design heights of coastal protection strategies in this class of financial decision‐making frameworks. They follow a “decision‐centered” approach (e.g., Ranger et al., 2013) because their outcomes are dependent on more than just SLR projections alone. Decision makers specify a tolerable level of risk (represented by a focal AAL), a protection strategy (elevation, levee, storm surge barrier, and coastal retreat), and assumptions regarding future AIS behavior (maximum AIS melt contribution and AIS stability). The latter is significant because decision‐making approaches that employ future projections of SLR may be appropriate when uncertainty can confidently be represented with a single PDF (e.g., near midcentury, when AIS melt uncertainty is well defined) but is insufficient when uncertainty is deep (e.g., late century AIS melt; Hall, 2007). While there is value in information to reduce deep uncertainty (e.g., constraining projections of AIS melt contributions; Dutton et al., 2015), until understanding improves regarding these aspects, approaches to flood protection design that rely on future distributions of relevant variables may require a multiprior approach to more accurately depict current states of knowledge.

## Supporting information



Supporting Information S1
