# Supplementary material for: A Flood Damage Allowance Framework for Coastal Protection With Deep Uncertainty in Sea Level Rise
Source: Earths Future. 2020 Mar 10;8(3):e2019EF001340. doi: 10.1029/2019EF001340 (PMC7375071; doi:10.1029/2019EF001340)
Supplement: Supplementary file 1 — Supporting Information S1 [file EFT2-8-e2019EF001340-s001.pdf]

Supporting Information for "A flood damage allowance framework for coastal protection with deep uncertainty in sea-level rise"

D.J. Rasmussen[1], Maya K. Buchanan[2], Robert E. Kopp[3,4], Michael Oppenheimer[1,5]

[1]Woodrow Wilson School of Public and International Affairs, Princeton University, Princeton, NJ, USA
[2]Climate Central, Princeton, NJ, USA
[3]Department of Earth & Planetary Sciences, Rutgers University, Piscataway, NJ, USA
[4]Institute of Earth, Ocean, and Atmospheric Sciences, Rutgers University, New Brunswick, NJ, USA
[5]Department of Geosciences, Princeton University, Princeton, NJ, USA

A Supplementary Methods

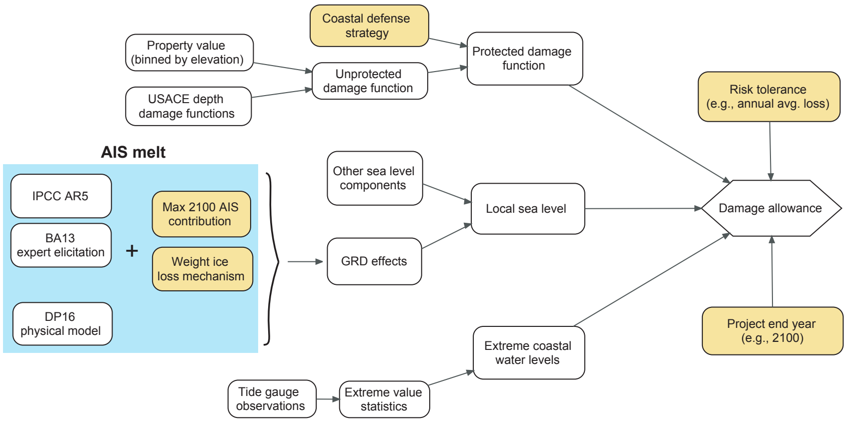

Figure A.1. Logical flow of sources of information used in damage allowance calculation. Yellow shading indicates points where decision maker input is required (Fig. 3). AIS is Antarctic Ice Sheet; USACE is U.S. Army Corps of Engineers; BA13 is Bamber and Aspinall (2013); DP16 is Deconto and Pollard (2016); GRD are gravitational, rotational, and deformational effects; "Other sea level components" includes land water storage, Greenland ice sheet melt, glacier ice melt, ocean dynamics, and non-climatic background changes, such as human-induced subsidence.

A.1 Modeling the frequency of extreme sea levels

We model the spatial extent of ESLs throughout Manhattan using the "bathtub" approach. This approach employs a 0.3 m (1 ft) horizontal resolution light detection and ranging (LiDAR)-derived digital elevation model (DEM[1]; Fig. A.2) and empirical estimates of the likelihood of ESLs at a single, long-standing tide gauge (1920–2014), located at the Battery in lower Manhattan. We calculate daily maximum sea levels from quality-controlled tide gauge records from the University of Hawaii Sea Level Center[2]. These tide gauge observations are de-trended to remove the effect of SLR and then referenced

Corresponding author: D.J. Rasmussen, `dj.rasmussen@princeton.edu`

[1] https://data.cityofnewyork.us/City-Government/1-foot-Digital-Elevation-Model-DEM-/dpc8-z3jc
[2] retrieved from: https://uhslc.soest.hawaii.edu, June 2017; Caldwell, Merrifield, and Thompson (2015)

to a common datum, mean higher high water (MHHW)[3]. The water levels at the Battery tide gauge are spatially extrapolated throughout Manhattan while considering 1) the topography of the study region and 2) an assumed 1-m high bulkhead (above MHHW) around the entire borough that serves as an estimate of the current level of coastal flood protection (Colle et al., 2008). We acknowledge that the bathtub approach is likely to be a poor estimator of the spatial ESL variation in this study region. The frequency of extreme water levels is known to differ throughout the New York-New Jersey Harbor Estuary due to hydrological factors such as varying coastal bathymetry and shoreline topography. A three-dimensional hydrodynamic model may more accurately represent these local characteristics (e.g., Aerts et al., 2014; Patrick, Solecki, Gornitz, Orton, & Blumberg, 2019). In any case, we note that the our estimated 100-yr flood extents (Fig. 4) are comparable to the preliminary flood insurance rate maps for New York City presented in Patrick et al. (2019).

We estimate the return periods of ESLs of various heights at the Battery tide gauge using extreme value theory, a statistical extrapolation method that fits an extreme value distribution to empirical data to estimate the likelihood of events too rare to appear in an observational record (e.g., determining the height of the 100-yr ESL from a 30-yr tide gauge record). Various extreme value distributions and approaches to implement them have been proposed (e.g., Coles, 2001b), but in the case of ESL estimation there currently is not an agreed upon "best approach". Depending on the specific project goals, a particular extreme value modeling strategy may be preferred over another (e.g., the length of observation record; Wahl et al., 2017).

Following previous studies (Buchanan et al., 2016; Rasmussen et al., 2018; Tebaldi, Strauss, & Zervas, 2012), we estimate the annual probability of ESLs $f(z)$ at the Battery tide gauge using a generalized Pareto distribution (GPD; Coles, 2001a, 2001b). The GPD has the advantage over other generalized extreme value models in that 1) it can accommodate sub-annual observations and 2) its third parameter (i.e., shape) allows for added flexibility to take on different shapes in log-linear space depending on the characteristics of the underlying data. The GPD is given by:

$$f_{(\xi,\mu,\sigma)}(z) = \frac{1}{\sigma}\left(1 + \frac{\xi(z-\mu)}{\sigma}\right)^{\left(-\frac{1}{\xi}-1\right)}, \tag{A.1}$$

for $z \geqslant \mu$ when $\xi \geqslant 0$, and $\mu \leqslant z \leqslant \mu - \sigma/\xi$ when $\xi < 0$.

The GPD parameters are the following: the shape parameter (ξ) governs the curvature and upward statistical limit of the ESL probability distribution function (PDF) and embodies the local coastal storm climate, the scale parameter (σ) characterizes the annual variability in the maxima of tides and storm surges, and the location parameter (μ) is the threshold water-level above which return levels are estimated with the GPD—here the 99th percentile of daily maximum sea levels, which is generally above the highest seasonal tide, balances the bias-variance trade-off in the GPD parameter estimation (Tebaldi et al., 2012) and has been found to perform well at global scales (Wahl et al., 2017). Daily maximum sea levels above the 99th percentile are de-clustered to meet the statistical independence assumption of the GPD. The GPD parameters are estimated using the method of maximum likelihood. Uncertainty in the GPD parameters is calculated from their estimated covariance matrix and is sampled using Latin hypercube sampling of 1000 normally distributed GPD parameter pairs. The GPD parameters used are given in Table A.1. The historical flood return curve at the Battery tide gauge is presented in Fig. 1A. While we estimate flood heights up to the frequency of 1 in 10,000, we caution in using

[3] Here defined as the average level of high tide over the last 19-years in the tide gauge record, which is different from the current U.S. National Tidal Datum Epoch of 1983–2001.

any extrapolations exceeding four times the length of the record (Pugh & Woodworth, 2014). Increases in SLR are accounted for linearly, as the nonlinear effect of SLR on ESL height has been determined to be very small for the Battery (Lin, Emanuel, Oppenheimer, & Vanmarcke, 2012).

Site	Lat	Lon	Uhawaii ID	Start	End	λ	μ (m)	ξ	σ
Battery	40.7	-74.15	745a	1920	2014	2.63	0.51	0.19 (0.05, 0.33)	0.13 (0.10, 0.15)

Table A.1. Generalized Pareto distribution (GPD) parameters estimated for the Battery tide gauge in Manhattan (New York City; Sec. A.1). The GPD threshold (μ) is given as meters above mean higher high water (MHHW). Both the shape (ξ) and scale (σ) parameters of the GPD are given as 50th (5th/95th) percentiles as estimate from the Latin hypercube sampling. Parameters are estimated using the method of maximum likelihood.

A.2 Manhattan terrain elevation map

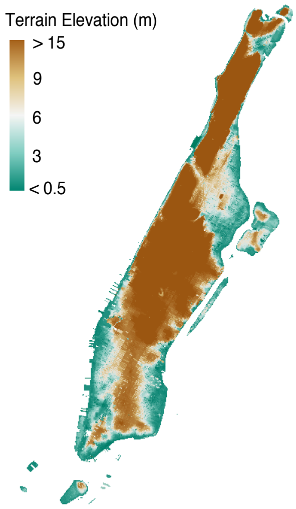

Figure A.2. Terrain elevation map for Manhattan (meters above the North American Vertical Datum of 1988) using a 0.3 m horizontal resolution light detection and ranging (LiDAR)-derived digital elevation model (DEM) from the City of New York[5]

A.3 Sea-level rise projections

Probabilistic, time-varying, local relative sea level (RSL) projections for Manhattan (modeled at the Battery tide gauge[6]) are taken from the component-based studies of Kopp et al. (2014) and Kopp et al. (2017). Both frameworks are identical except in how they model Antarctic ice sheet (AIS) contributions. Kopp et al. (2014) combines the Intergovernmental Panel on Climate Change's Fifth Assessment Report likely range projections of ice sheet dynamics and surface mass balance (table 13.5 in Church, Clark, et al., 2013) and tail shape information from the expert elicitation of total ice sheet mass

[6] https://tidesandcurrents.noaa.gov/stationhome.html?id=8518750

loss from Bamber and Aspinall (2013), while Kopp et al. (2017) implement a limited ensemble of physical AIS simulations from Deconto and Pollard (2016). Deconto and Pollard (2016) includes two glaciological processes previously not accounted for in other continental scale models that can rapidly increase ice-sheet mass loss (marine ice-sheet hydrofracturing and marine ice-cliff instability; Pollard, DeConto, & Alley, 2015). The simulations from Deconto and Pollard (2016) do not sample the full model parameter space, as such they do not provide a probabilistic assessment of future AIS behavior (Edwards et al., 2019; Kopp et al., 2017). Nonetheless, they previously have been implemented in probabilistic projection frameworks (Bakker et al., 2017; Le Bars, Drijfhout, & de Vries, 2017). Probability distributions of local RSL are produced using 10,000 Latin hypercube samples of individual sea level component contributions. Each probability distribution is conditional on either the high greenhouse gas (GHG) emission scenario of representative concentration pathway (RCP) 8.5 or the strong GHG reduction scenario of RCP2.6 (Van Vuuren et al., 2011). The general circulation model output used to generate the steric and glacial ice melt sea level components for each RCP scenario are given in Table S2 in the Supporting Information of Kopp et al. (2014).

A.4 Extreme sea level damage model

We model the annual average loss (AAL) due to extreme sea level (ESL) damage as the average loss (insured and uninsured) of all modeled ESL damage events $D(z)$, weighted by the annual probability of occurrence $f(z)$. This can mathematically be written as,

$$\mathbb{E}[D(z)] = \int_z D(z) f(z)\,\mathrm{d}z. \tag{A.2}$$

While this is a one-dimensional model (vertical direction only), we note that spatial variation in coastal protection and ESL event frequency could be accommodated using a three-dimensional damage model where the ESL parameter (z) is a vector that also varies spatially. For example, this could be done using a three-dimensional hydrodynamic model (e.g., Aerts et al., 2014; Patrick et al., 2019). Other limitations to our flood damage modeling approach that could impact results are noted throughout the methodological overview given below.

A.5 Modeling damages from extreme sea levels

Following the methodology from Diaz (2016), we construct a one-dimensional (z-direction), aggregate ESL damage function for Manhattan by integrating damages from the lowest unprotected elevation e_{min} to an ESL height z using:

$$\mathrm{D(z)} = \int_{e_{min}}^{z} p(e) \cdot \phi(z - e)\mathrm{d}e \tag{A.3}$$

where $p(e)$ is the total tax assessed value of all buildings at the estimated first floor elevation e from the NYC Department of City Planning (Fig. A.3; NYC Planning, 2018)[7], z is the ESL height, and $\phi(z{-}e)$ is an aggregate inundation depth-damage function for Manhattan that relates the flood height $(z{-}e)$ to damage as a fraction of the total tax assessed building value (see Sec. A.6). The first floor structure elevation was estimated in 0.1 m increments starting from 0 m above the North American Vertical Datum of 1988 (NAVD88) using a 0.3 m horizontal resolution LiDAR-derived digital elevation model

[7] Note that the NYC Department of City Planning makes available both tax assessed building value and combined tax assessed building and property value. We assume that floods only damage structures and not the land itself.

(DEM) from the City of New York[8]. Other covariates that may cause damage, such as wind gusts, waves, and precipitation, are not included. Also, not included in our damage accounting is the loss of human life, damage to infrastructure (both above and below ground), the value lost from permanently inundated lands, and indirect damage effects such as business interruption. In order to simplify our analysis and to isolate the impact of sea-level rise on changing risk, we assume that the population and distribution of property within Manhattan remains fixed in time. We acknowledge that this assumption may not be realistic. For instance, the NYC Department of City Planning has projected a 3.9% increase in the population of Manhattan over 2020–2050 (NYMTC, 2015). Population projections for Manhattan after mid-century are not available, but other flood damage mitigation studies have argued that the population and building stock of NYC will become relatively stable after mid century (Aerts et al., 2014).

Figure 1B shows the damage function. The damage function is multiplied by the ESL probability distribution (Sec. A.1) to give a probability distribution of flood damages (Fig. 1C). We estimate that the current AAL for Manhattan is \$0.10 billion/yr. Comparison with other studies is difficult due to differences in geographic scope and building stock. Nonetheless, Aerts et al. (2014) and Houser et al. (2015) found an AAL of \$0.18 and \$0.53 billion/yr for all of NYC, respectively, and Hallegatte et al. (2013) found an AAL of \$0.79 billion/yr for the entire NYC-Newark, New Jersey region (all values given in 2017 US\$).

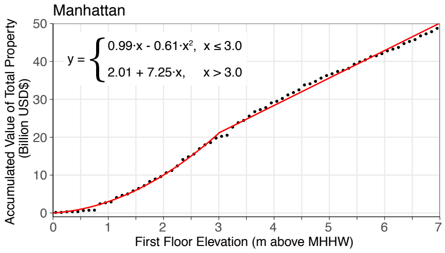

Figure A.3. Accumulated tax-assessed value of Manhattan property (building only, land excluded; billion USD\$) by first floor building elevation [meters above mean higher high water (MHHW); black circles] and piece-wise fit using a quadratic function below 3 m and linear function above 3 m (red line). Property data is from the New York City Department of City Planning (NYC Planning, 2018).

A.6 Depth-damage functions

Depth-damage functions describe relationships between flood-depth and levels of structure damage. They are often constructed from post-disaster assessments of actual damage to various classes of structures (e.g., one-story residential homes with basements, schools, shopping centers). Depth-damage functions are a commonly used approach for modeling flood damage given flood depth (e.g., FEMA, 2018). Depth-damage functions are simplistic representations of the relationship between floods and structure damage in part because they only consider one characteristic of floods—depth. They do not con-

[8] https://data.cityofnewyork.us/City-Government/1-foot-Digital-Elevation-Model-DEM-/dpc8-z3jc

sider other potentially important flood characteristics that may cause more damage, such as flood velocity, wave height, and flood duration (Merz, Kreibich, Schwarze, & Thieken, 2010).

In this study, we employ structure-specific depth-damage functions constructed from expert elicitation (USACE, 2015). While Manhattan is comprised of several thousand varieties of structures, each potentially having a unique relationship between inundation depth and damage, we simplify by assuming only three distinct classes of buildings in the study region of Manhattan (New York City). New York City property tax assessments indicate that Manhattan is comprised of roughly 95 percent residential and 5 percent commercial (NYC Department of Finance, 2018). Accordingly, we reduce the complexity of building type to three inundation depth-damage functions that represent these classes of buildings, high-rises with basements (95 percent of Manhattan) and two-story residences with basements (5 percent of Manhattan). We note that this simplification could have a significant impact on aggregate flood damage estimates for Manhattan.

The depth-damage function for an urban high-rise ($\phi_{hrise}(z-e)$) is shown in SI Fig. A.5, and the least-squares fit is given by:

$$\phi_{hrise}(z-e) = \begin{cases} 0.142 + 0.0541 \cdot (z-e) - 0.00368 \cdot (z-e)^2 - 0.00133 \cdot (z-e)^3, & \text{if } z > e \\ 0, & \text{otherwise} \end{cases}$$

where z is the height of the extreme water level, e is the first-floor elevation of the structure, and $z-e$ is the flood height. The depth-damage function for a two-story residential structure with a basement ($\phi_{res}(z-e)$) is shown in SI Fig. A.4, and the least-squares fit is given by,

$$\phi_{res}(z-e) = \begin{cases} 0.18 + 0.178 \cdot (z-e) + 0.0233 \cdot (z-e)^2 - 0.00778 \cdot (z-e)^3, & \text{if } z > e \\ 0, & \text{otherwise} \end{cases}$$

An aggregate depth-damage function for Manhattan is constructed using the weighted average of the equations for each building class,

$$\phi(z-e) = 0.95 \cdot \phi_{hrise}(z-e)) + 0.05 \cdot \phi_{res}(z-e).$$

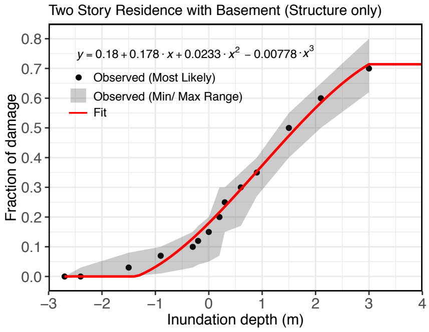

Figure A.4. Observed depth-damage relationship for two story residences with basements (structure only) from USACE (2015). The contents of the structure are not included. A 3rd-order polynomial is fit through the observed (most likely) values (red line).

Two Story Residence with Basement (Structure only)

Depth (ft)	Depth (m)	Min	Most Likely	Max
-9.0	-2.7	0.000	0.000	0.000
-8.0	-2.4	0.000	0.000	0.030
-5.0	-1.5	0.000	0.030	0.080
-3.0	-0.9	0.010	0.070	0.100
-1.0	-0.3	0.030	0.100	0.150
-0.5	-0.2	0.040	0.120	0.170
0.0	0.0	0.050	0.150	0.200
0.5	0.2	0.070	0.200	0.300
1.0	0.3	0.150	0.250	0.300
2.0	0.6	0.170	0.300	0.350
3.0	0.9	0.270	0.350	0.400
5.0	1.5	0.400	0.500	0.550
7.0	2.1	0.500	0.600	0.650
10.0	3.0	0.620	0.700	0.800

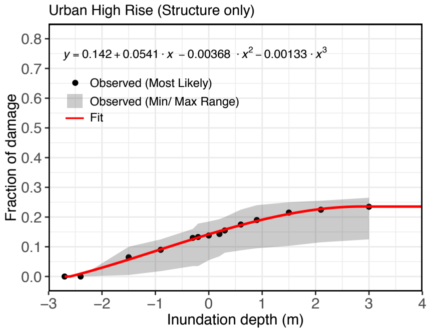

Figure A.5. Observed depth-damage relationship for an urban high-rise (structure only) from USACE (2015). A 3rd-order polynomial is fit through the observed (most likely) values (red line).

Urban High Rise (Structure only)

Depth (ft)	Depth (m)	Min	Most Likely	Max
-9.0	-2.7	0.000	0.000	0.000
-8.0	-2.4	0.000	0.000	0.000
-5.0	-1.5	0.005	0.065	0.100
-3.0	-0.9	0.018	0.090	0.125
-1.0	-0.3	0.035	0.130	0.160
-0.5	-0.2	0.035	0.133	0.178
0.0	0.0	0.055	0.138	0.185
0.5	0.2	0.068	0.143	0.193
1.0	0.3	0.080	0.155	0.200
2.0	0.6	0.088	0.175	0.225
3.0	0.9	0.095	0.190	0.240
5.0	1.5	0.103	0.215	0.250
7.0	2.1	0.115	0.225	0.255
10.0	3.0	0.125	0.235	0.265

A.7 Returning hazard allowances from the damage allowance framework

We show how Eq. 2 can return the traditional hazard allowance (Eq. 1). If $D^*(z)$ is given by $D(z-A)$, then Eq. 2 becomes,

$$\int_z \int_\Delta D(z-A) f(z-\Delta) P(\Delta)\,\mathrm{d}\Delta\,\mathrm{d}z = \int_z D(z) f(z)\,\mathrm{d}z, \tag{A.4}$$

While Eq. A.4 is appropriate for estimating the adjustment to maintain the overall risk, adjustments can be calculated for specific AEPs. If z^* is the surge height of the current AEP event then,

$$\int_{z^*}^{\infty} \int_\Delta D(z-A) f(z-\Delta) P(\Delta)\,\mathrm{d}\Delta\,\mathrm{d}z = \int_{z^*}^{\infty} D(z) f(z)\,\mathrm{d}z, \tag{A.5}$$

integrating over z gives,

$$\int_\Delta D(z^*-A) \mathrm{F}(z^*-\Delta) P(\Delta)\,\mathrm{d}\Delta \ = D(z^*) \mathrm{F}(z^*), \tag{A.6}$$

where F is the cumulative distribution function, which is related to the expected number of exceedances in a given year N by $N = -\log(\mathrm{F})$ (Hunter, 2012; Pugh, 1996),

$$\int_\Delta D(z^*-A) N(z^*-\Delta) P(\Delta)\,\mathrm{d}\Delta \ = D(z^*) N(z^*). \tag{A.7}$$

where $N(z^*)$ is the number of expected flood events per year of height z^* without SLR. Integrating over $P(\Delta)$, the SLR PDF, gives

$$D(z^*-A) N_e(z^*-\Delta) = D(z^*) N(z^*), \tag{A.8}$$

where $N_e(z^*)$ is the number of expected flood events per year of height z^* after considering the SLR PDF. The traditional allowances for physical flood heights (e.g., Buchanan et al., 2016; Hunter, 2012) can be returned if $D(z)$ is the function,

$$D[z] = \begin{cases} 0, & z < z^*, \\ 1, & z \geq z^*. \end{cases} \tag{A.9}$$

Substituting Eq. A.9 in Eq. A.5 gives,

$$\int_{z^*+A}^{\infty} \int_\Delta f(z-\Delta) P(\Delta)\,\mathrm{d}\Delta\,\mathrm{d}z = \int_{z^*}^{\infty} f(z)\,\mathrm{d}z, \tag{A.10}$$

and then integrating over all possible surges z gives,

$$\int_\Delta N(z^*-\Delta+A) P(\Delta)\,\mathrm{d}\Delta = N(z^*), \tag{A.11}$$

where $N(z^*)$ is the average number of flood exceedance events of height z^* per year. After integrating over the SLR probability distribution the equality becomes,

$$N_e(z^*+A) = N(z^*), \tag{A.12}$$

where $N_e(z)$ returns the average number of flood exceedance events of height z per year after including uncertain SLR. The allowance height A must be solved for numerically.

A.8 Elevation of all structures by same height

While not used for Manhattan due to the impracticality of elevating high-rises, we present an method for elevation all structures within the damage function (Fig. A.6). If A is the vertical height that all structures would need to be elevated in order to maintain the current AAL under uncertain sea-level rise and if α is the fraction of assets [0,1] that have elevated by A (i.e., the elevation compliance), then the protected damage function is:

$$D^*(z, A) = \underbrace{\alpha D(z - A)}_{\text{Damage to elevated structures}} + \underbrace{(1 - \alpha) D(A).}_{\text{Damage to non-elevated structures}} \tag{A.13}$$

The elevation of all structures is mathematically represented as a horizontal shift of the "unprotected" damage function by A to represent the uniform elevation of all assets by A.

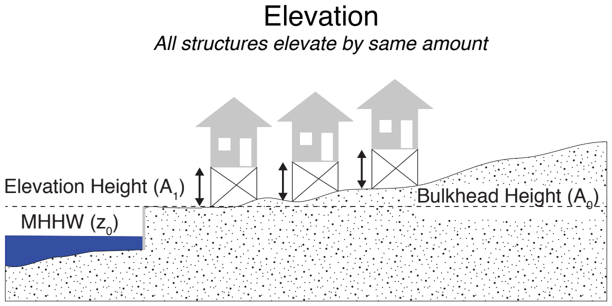

Figure A.6. Schematic illustrating an elevation flood defense strategy (all structures) for an arbitrary design height (A_1).

A.9 Hazard allowances: instantaneous vs. average annual design life

Two types of hazard allowances have been proposed in the literature: 1) instantaneous and 2) average annual design life. Instantaneous hazard allowances are designed for maintaining a target level of risk in the final year of a project design life (traditionally the expected number of ESL exceedances). In the preceding years, the average level of risk protection would be above the target. On the other hand, the average annual design life hazard allowance (not explored in this study), maintains the average risk over the lifetime of a project by providing greater protection than prescribed during the early years of the design life and less protection than prescribed at the end of the design life (Buchanan et al., 2016).

A.10 Combined flood protection strategy approach

A multi-strategy approach to flood defenses may provide an added level of safety through redundancy. For example, if a single levee fails, the area behind the levee is impacted. A second line of defense could compensate for failures of the first. If multiple

strategies are employed, users could either assign a fraction of the total risk target to mitigate for each strategy or specify a damage allowance for all but one mitigation strategy and then solve for the unknown damage allowance. For example, if a user desires to maintain the current AAL using both coastal retreat and a levee, they may choose to retreat coastal assets below a pre-determined elevation A_1 that is also the base of the levee (e.g., $A_1 = 1.0$ m) and then solve for the height of the levee A_2 (Fig. B.9A). This can mathematically be described by:

$$\underbrace{\int_{z_{min}}^{A_1} \int_{\Delta} D_r^*(z) f(z-\Delta) P(\Delta)\,\mathrm{d}\Delta\,\mathrm{d}z}_{\text{Damages below retreat elevation}} + \underbrace{\int_{A_1}^{\infty} \int_{\Delta} D_l^*(z) f(z-\Delta) P(\Delta)\,\mathrm{d}\Delta\,\mathrm{d}z}_{\text{Damages from levee failure and overtopping}} = \underbrace{\int_{z_{min}}^{\infty} D(z) f(z)\,\mathrm{d}z}_{\text{Current AAL}}. \tag{A.14}$$

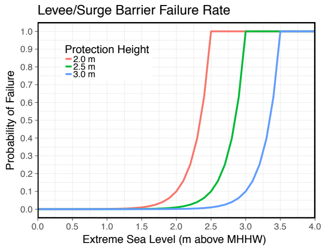

Figure A.7. Fragility curves showing the relationship between structural loading on a levee or storm surge barrier from extreme sea levels (meters above mean higher high water [MHHW]) and the conditional probability of structural failure of the levee or storm surge barrier for protection design heights of 2.0 m (red), 2.5 m (green), 3.0 m (blue), all with 0.5 m of freeboard above the design height. For all, the structural failure rate for extreme sea levels at the design height is 0.10.

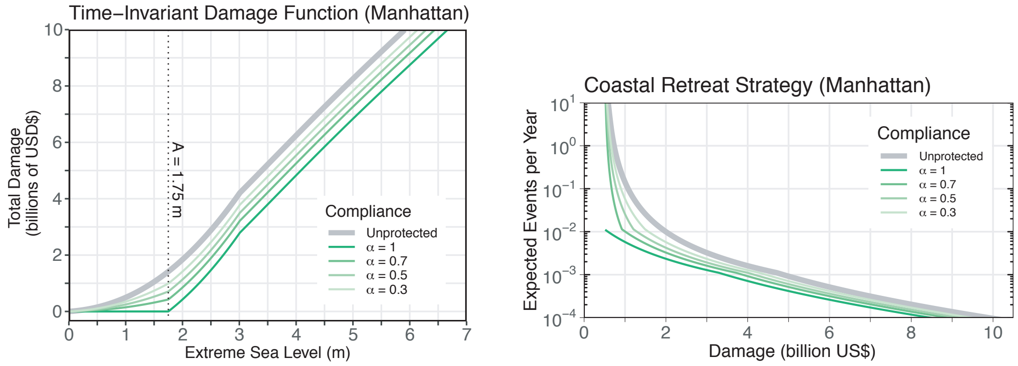

Figure A.8. **(Left)** Expected number of damage events per year for Manhattan assuming no change in protection strategy (grey line) and using an elevation strategy (1.75 m) with various levels of compliance (α; red lines) **(Right)** As for Left, but for a coastal retreat strategy (1.75 m; green lines)

A.11 Probability box construction

Our approach to constructing a probability box ('p-box') is presented in Section 2.2, but is expanded with more details here. In order to keep the p-box boundaries from overlapping, we arbitrarily truncate the maximum AIS contribution from Kopp et al. (2014) at 1.75 m (relative to 2000), the highest predicted AIS contributions from Deconto and Pollard (2016). This limits the maximum 2100 GMSL projection below 3.5 m (relative to 2000). We note that this truncation is arbitrary and is used only for the purpose of illustrating the p-box approach to dealing with deep uncertainty. The truncation of the AIS contribution about the 1.75 m limit could impact results in a significant way, but is not investigated here. There currently is no consensus upper limit for 2100 GMSL or AIS contributions. The 5/95th percentile ranges of GMSL from Kopp et al. (2014) and Kopp et al. (2017) roughly bound either the 17/83 or 5/95 end-of-century ranges from current published RCP8.5 GMSL projections surveyed in Horton et al. (2018), but not those for RCP2.6.

Flood allowances and ESL return curves that consider the full probability distribution of sea level projections have been shown to be sensitive to upper-bound estimates of AIS ice mass loss in the second half of the 21st century (Buchanan et al., 2016; Rasmussen et al., 2018; Slangen et al., 2017). As such, we use a parameter that sets the truncation of the upper tail of the 2100 AIS contribution distribution (AIS_{max}). Specifically, we use AIS_{max} values of 0.25, 0.5, and 1.0 m, which are in-line with the range of published end-of-century AIS melt estimates (e.g., Table 2 in Cozannet et al., 2017), as well as limits of 1.5 m and 1.75 m, which are upper-end estimates from Deconto and Pollard (2016). An additional parameter weighs contributions from the projections that bound the p-box ($\beta_c \in [0, 1]$). When there is greater confidence of AIS collapse (i.e., larger values of β_c), more weight is given to the Kopp et al. (2017) projections, which include faster ice mass loss and greater AIS contributions to GMSL in the second half of the 21st century (via marine ice-sheet hydrofracturing and ice-cliff collapse; Pollard et al., 2015), relative to Kopp et al. (2014). Note that a value of zero for β_c does not imply a scenario in which there is zero probability of AIS collapse initiation, nor does a value of one for β_c imply certainty in AIS collapse initiation. β_c simply corresponds to the relative likelihoods of AIS collapse initiation before 2100. The effective probability distribution $\tilde{P}$ at time t is given by:

$$\tilde{P}(\beta_c, \text{AIS}_{max}, t) = \beta_c P_{high}(\text{AIS}_{max}, t) + (1 - \beta_c) P_{low}(\text{AIS}_{max}, t), \tag{A.15}$$

where $P_{low}(\Delta, t)$ and $P_{high}(\Delta, t)$ are the minimum and maximum projections at each point in the CDFs from Kopp et al. (2014) and Kopp et al. (2017). All the SLR projection scenarios used in this study are highlighted in Table B.1.

B Supplemental Results, Figures and Tables

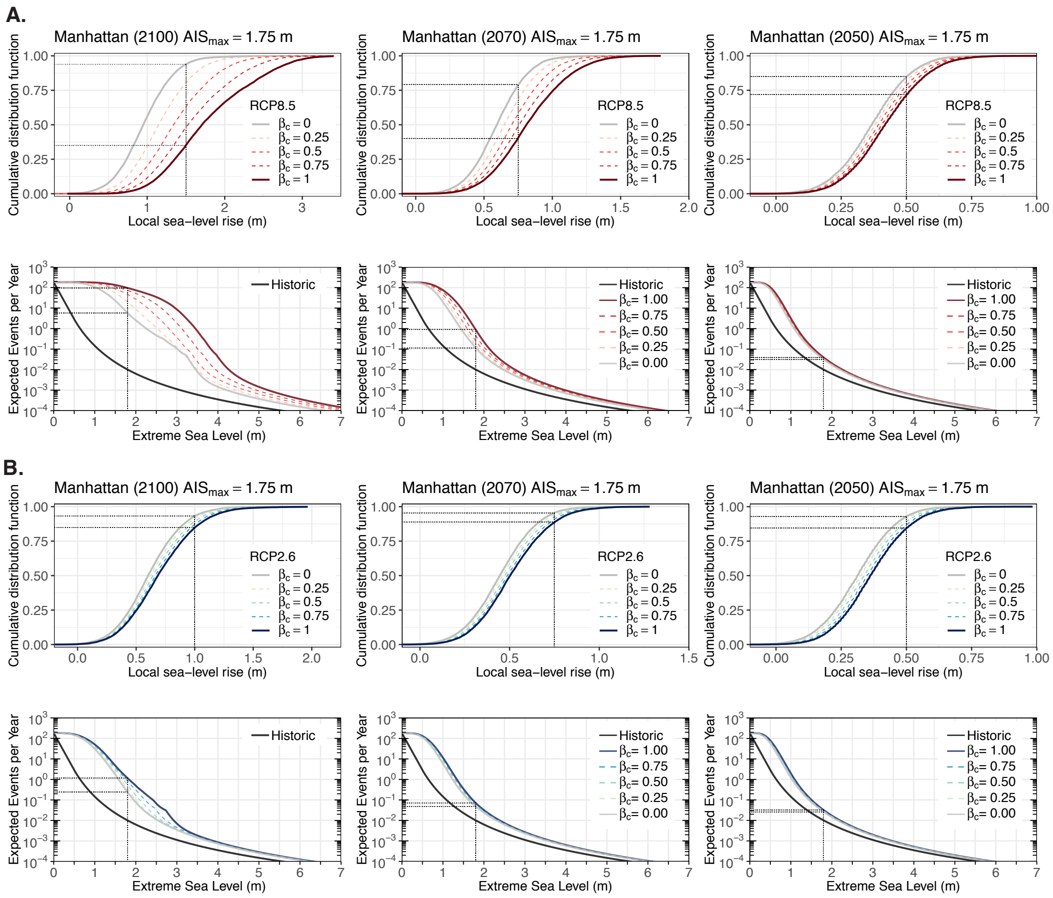

Figure B.1. **A.** Top: Probability boxes ('p-boxes'; solid lines) for 2100 (left), 2070 (center), and 2050 (right) local sea-level rise (SLR) in Manhattan (located at the Battery tide gauge) under the representative concentration pathway (RCP) 8.5 climate forcing scenario. Effective cumulative distribution functions of local SLR (dashed lines) are generated within each p-box by averaging the edges using weights ($\beta_c \in [0,1]$) that reflect a user's belief of AIS collapse initiation within the 21st century (higher values reflect higher likelihood of collapse) and by constraining the maximum possible 2100 Antarctic Ice Sheet (AIS) melt (AIS_{max}, relative to 2000; here, 1.75 m; Sec. 2.2). The black dotted lines highlight the probability of exceeding 1.5 m, 1.0 m, or 0.5 m of local SLR (1−CDF) under different assumptions of AIS collapse initiation (i.e., values of β_c). Bottom: extreme sea level (ESL) event return curves for Manhattan showing the relationship between the expected number of ESL events per year and ESL height (meters above mean higher high water) for: 1) historical sea levels (black curve) and 2) the year 2100, 2070, and 2050 (RCP8.5) for different values of β_c. All curves incorporate generalized Pareto distribution (GPD) parameter uncertainty (Sec. A.1) and the future return curves additionally incorporate local SLR projection uncertainty by integrating across the entire local SLR probability distribution. The black dotted lines highlight the annual expected number of historically experienced 100-yr ESL events under different values of β_c. **B.** As for A, but for RCP2.6.

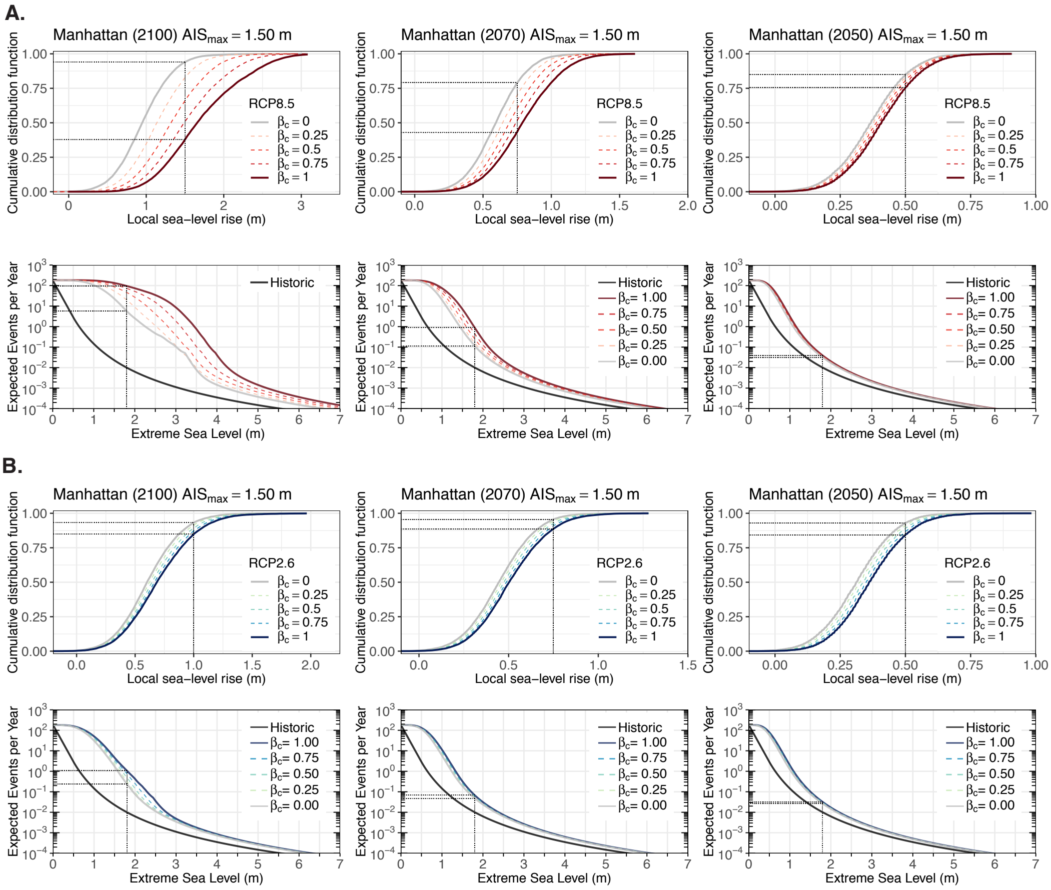

Figure B.2. **A.** Top: Probability boxes ('p-boxes'; solid lines) for 2100 (left), 2070 (center), and 2050 (right) local sea-level rise (SLR) in Manhattan (located at the Battery tide gauge) under the representative concentration pathway (RCP) 8.5 climate forcing scenario. Effective cumulative distribution functions of local SLR (dashed lines) are generated within each p-box by averaging the edges using weights ($\beta_c \in [0,1]$) that reflect a user's belief of AIS collapse initiation within the 21st century (higher values reflect higher likelihood of collapse) and by constraining the maximum possible 2100 Antarctic Ice Sheet (AIS) melt (AIS$_{max}$, relative to 2000; here, 1.5 m; Sec. 2.2). The black dotted lines highlight the probability of exceeding 1.5 m, 1.0 m, 0.75 m, or 0.5 m of local SLR (1−CDF) under different assumptions of AIS collapse initiation (i.e., values of β_c). Bottom: extreme sea level (ESL) event return curves for Manhattan showing the relationship between the expected number of ESL events per year and ESL height (meters above mean higher high water) for: 1) historical sea levels (black curve) and 2) the year 2100, 2070, and 2050 (RCP8.5) for different values of β_c. All curves incorporate generalized Pareto distribution (GPD) parameter uncertainty (Sec. A.1) and the future return curves additionally incorporate local SLR projection uncertainty by integrating across the entire local SLR probability distribution. The black dotted lines highlight the annual expected number of historically experienced 100-yr ESL events under different values of β_c. **B.** As for A, but for RCP2.6.

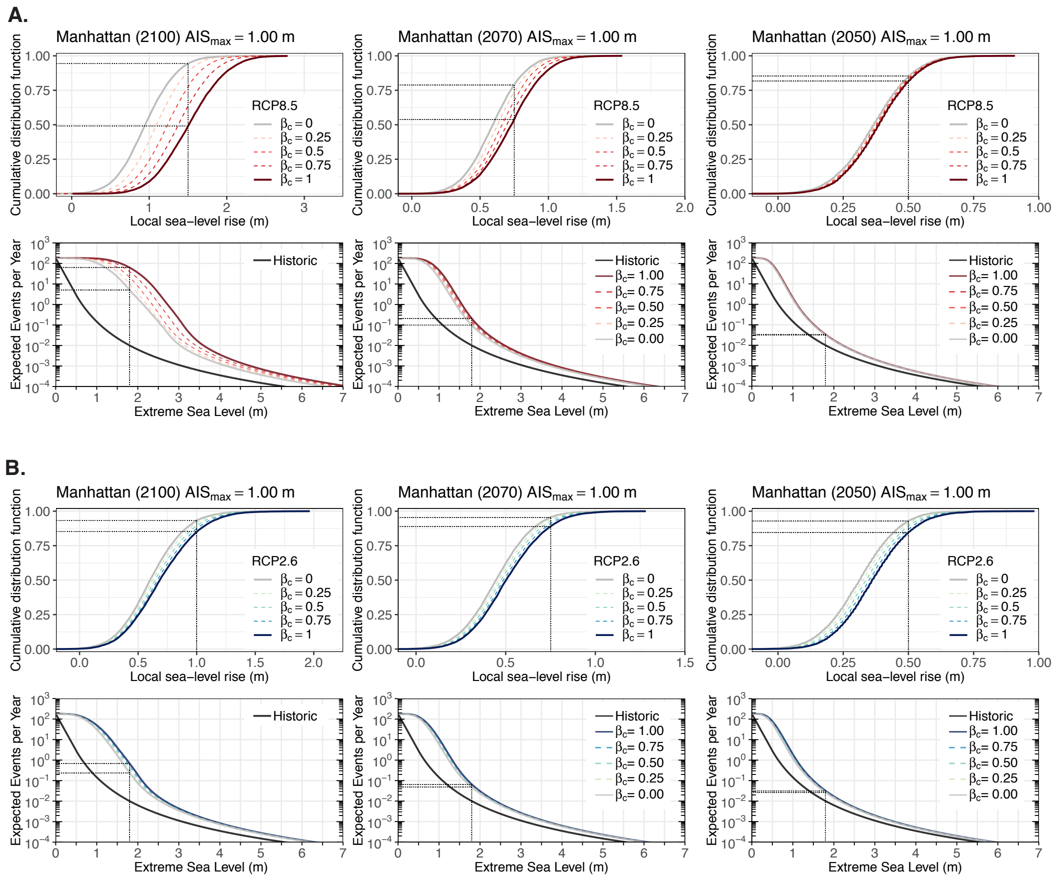

Figure B.3. **A.** Top: Probability boxes ('p-boxes'; solid lines) for 2100 (left), 2070 (center), and 2050 (right) local sea-level rise (SLR) in Manhattan (located at the Battery tide gauge) under the representative concentration pathway (RCP) 8.5 climate forcing scenario. Effective cumulative distribution functions of local SLR (dashed lines) are generated within each p-box by averaging the edges using weights ($\beta_c \in [0,1]$) that reflect a user's belief of AIS collapse initiation within the 21st century (higher values reflect higher likelihood of collapse) and by constraining the maximum possible 2100 Antarctic Ice Sheet (AIS) melt (AIS_{max}, relative to 2000; here, 1.0 m; Sec. 2.2). The black dotted lines highlight the probability of exceeding 1.5 m, 1.0 m, 0.75 m, or 0.5 m of local SLR (1−CDF) under different assumptions of AIS collapse initiation (i.e., values of β_c). Bottom: extreme sea level (ESL) event return curves for Manhattan showing the relationship between the expected number of ESL events per year and ESL height (meters above mean higher high water) for: 1) historical sea levels (black curve) and 2) the year 2100, 2070, and 2050 (RCP8.5) for different values of β_c. All curves incorporate generalized Pareto distribution (GPD) parameter uncertainty (Sec. A.1) and the future return curves additionally incorporate local SLR projection uncertainty by integrating across the entire local SLR probability distribution. The black dotted lines highlight the annual expected number of historically experienced 100-yr ESL events under different values of β_c. **B.** As for A, but for RCP2.6.

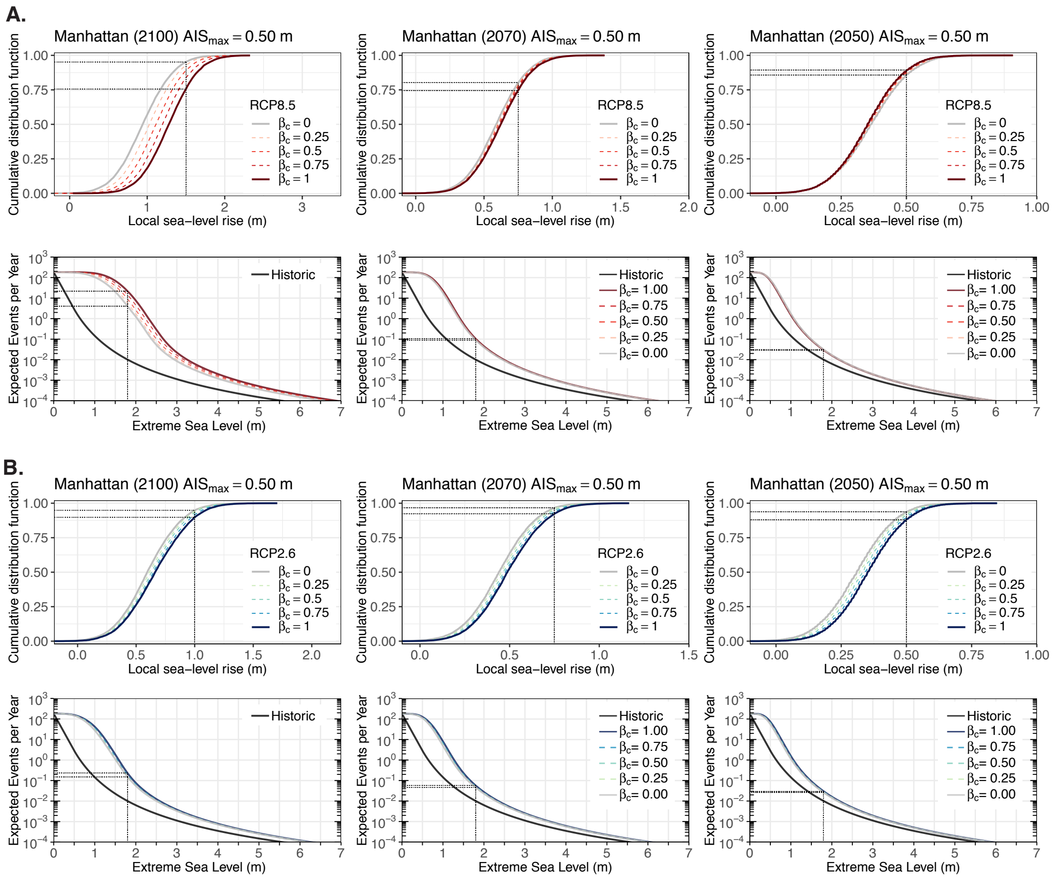

Figure B.4. **A.** Top: Probability boxes ('p-boxes'; solid lines) for 2100 (left), 2070 (center), and 2050 (right) local sea-level rise (SLR) in Manhattan (located at the Battery tide gauge) under the representative concentration pathway (RCP) 8.5 climate forcing scenario. Effective cumulative distribution functions of local SLR (dashed lines) are generated within each p-box by averaging the edges using weights ($\beta_c \in [0,1]$) that reflect a user's belief of AIS collapse initiation within the 21st century (higher values reflect higher likelihood of collapse) and by constraining the maximum possible 2100 Antarctic Ice Sheet (AIS) melt (AIS$_{max}$, relative to 2000; here, 0.5 m; Sec. 2.2). The black dotted lines highlight the probability of exceeding 1.5 m, 1.0 m, 0.75 m, or 0.5 m of local SLR (1−CDF) under different assumptions of AIS collapse initiation (i.e., values of β_c). Bottom: extreme sea level (ESL) event return curves for Manhattan showing the relationship between the expected number of ESL events per year and ESL height (meters above mean higher high water) for: 1) historical sea levels (black curve) and 2) the year 2100, 2070, and 2050 (RCP8.5) for different values of β_c. All curves incorporate generalized Pareto distribution (GPD) parameter uncertainty (Sec. A.1) and the future return curves additionally incorporate local SLR projection uncertainty by integrating across the entire local SLR probability distribution. The black dotted lines highlight the annual expected number of historically experienced 100-yr ESL events under different values of β_c. **B.** As for A, but for RCP2.6.

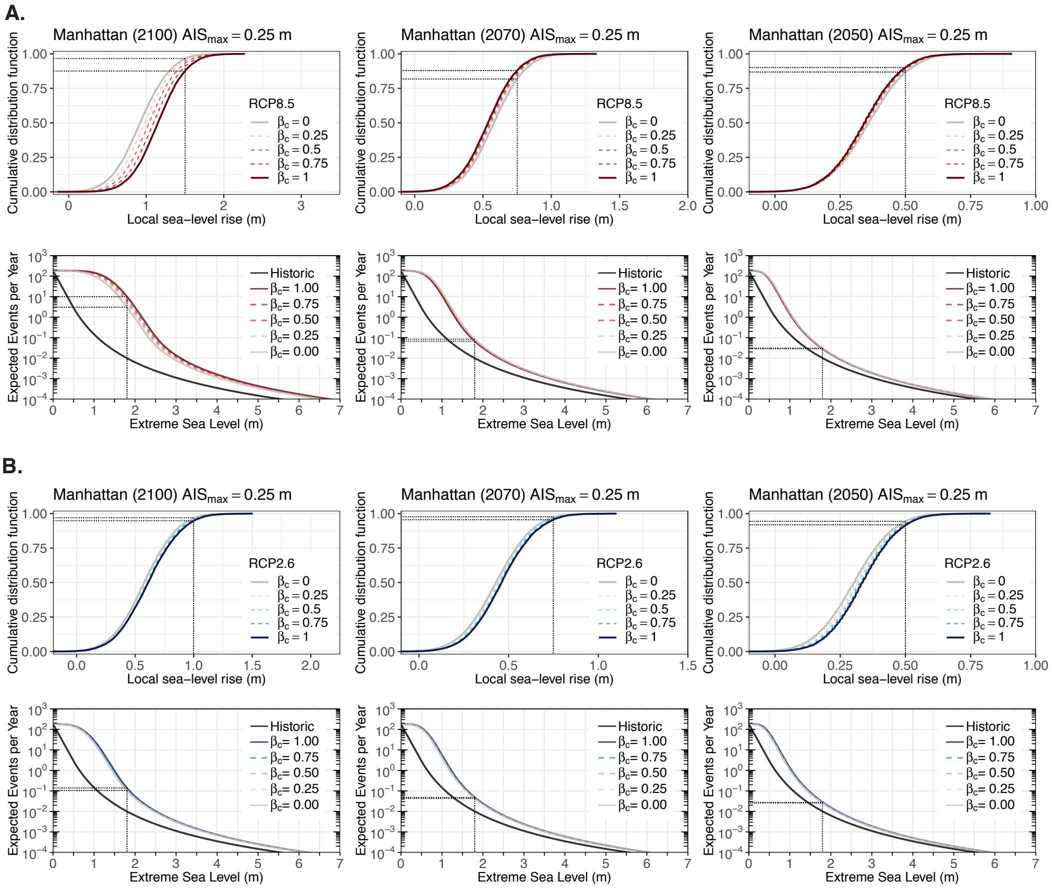

Figure B.5. **A.** Top: Probability boxes ('p-boxes'; solid lines) for 2100 (left), 2070 (center), and 2050 (right) local sea-level rise (SLR) in Manhattan (located at the Battery tide gauge) under the representative concentration pathway (RCP) 8.5 climate forcing scenario. Effective cumulative distribution functions of local SLR (dashed lines) are generated within each p-box by averaging the edges using weights ($\beta_c \in [0,1]$) that reflect a user's belief of AIS collapse initiation within the 21st century (higher values reflect higher likelihood of collapse) and by constraining the maximum possible 2100 Antarctic Ice Sheet (AIS) melt (AIS_{max}, relative to 2000; here, 0.25 m; Sec. 2.2). The black dotted lines highlight the probability of exceeding 1.5 m, 1.0 m, 0.75 m, or 0.5 m of local SLR ($1-$CDF) under different assumptions of AIS collapse initiation (i.e., values of β_c). Bottom: extreme sea level (ESL) event return curves for Manhattan showing the relationship between the expected number of ESL events per year and ESL height (meters above mean higher high water [MHHW]) for: 1) historical sea levels (black curve) and 2) the year 2100, 2070, and 2050 (RCP8.5) for different values of β_c. All curves incorporate generalized Pareto distribution (GPD) parameter uncertainty (Sec. A.1) and the future return curves additionally incorporate local SLR projection uncertainty by integrating across the entire local SLR probability distribution. The black dotted lines highlight the annual expected number of historically experienced 100-yr ESL events under different values of β_c. **B.** As for A, but for RCP2.6.

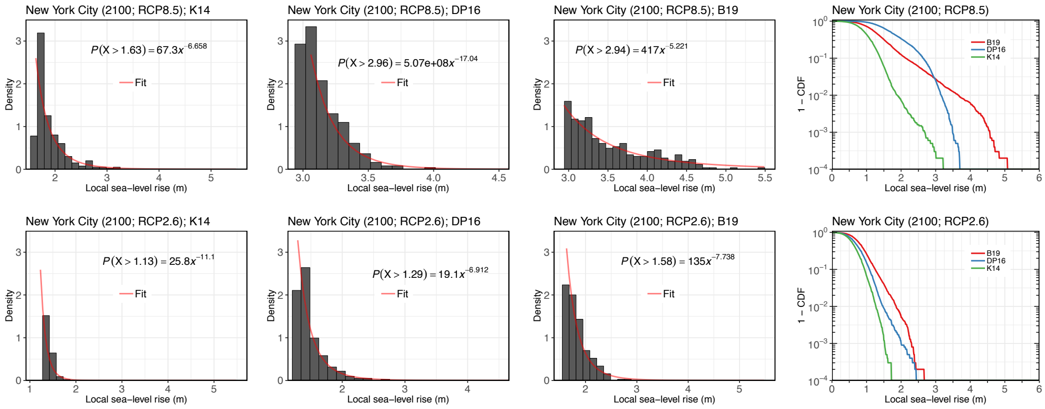

Figure B.6. **Top Row:** Density plot of local sea-level rise (SLR) samples (meters relative to 2000) above the 97th percentile for 2100 for New York City (RCP8.5) and a power function fit (red line). The smaller the absolute value of the exponent of the power function fit, the longer the tail of the SLR distribution. The SLR samples in each plot are from the probabilistic projections of Kopp et al. (2014)(K14), Deconto and Pollard (2016)(DP16), and Bamber et al. (2019)(B19). Far right plot is a survival function for 2100 local SLR (meters) for New York City from Bamber et al. (2019)(B19; red), Deconto and Pollard (2016)(DP16; blue), and Kopp et al. (2014)(K14; green). **Bottom Row:** As for Top Row, but for RCP2.6.

Table B.1. Future local sea-level rise (SLR) projections (meters; relative to 2000) at a tide gauge located at the Battery in lower Manhattan (New York City) for 2100, 2070, and 2050 under representative concentration pathway (RCP) 8.5 and RCP2.6 and for different assumptions regarding future Antarctic ice sheet (AIS) behavior (e.g, likelihood of AIS collapse [β_c] and maximum 2100 AIS contribution [AIS$_{max}$]). Values given are: expected (5th percentile–95th percentile).

Local Sea-Level Rise (m; relative to 2000)

RCP8.5		AIS$_{max}$					RCP2.6		AIS$_{max}$				
	β_c	1.75 m	1.5 m	1.0 m	0.5 m	0.25 m		β_c	1.75 m	1.5 m	1.0 m	0.5 m	0.25 m
2100							2100						
	1	1.8 (1.0–2.9)	1.7 (0.9–2.7)	1.5 (0.9–2.2)	1.3 (0.8–1.8)	1.2 (0.7–1.7)		1	0.7 (0.3–1.2)	0.7 (0.3–1.2)	0.7 (0.3–1.2)	0.7 (0.3–1.1)	0.6 (0.2–1.0)
	0.75	1.6 (0.8–2.5)	1.5 (0.8–2.3)	1.4 (0.8–2.0)	1.2 (0.7–1.7)	1.1 (0.6–1.6)		0.75	0.7 (0.3–1.2)	0.7 (0.3–1.2)	0.7 (0.3–1.2)	0.7 (0.3–1.1)	0.6 (0.2–1.0)
	0.5	1.4 (0.7–2.2)	1.3 (0.7–2.0)	1.2 (0.7–1.8)	1.1 (0.6–1.6)	1.0 (0.6–1.6)		0.5	0.7 (0.2–1.1)	0.7 (0.2–1.1)	0.7 (0.2–1.1)	0.6 (0.2–1.0)	0.6 (0.2–1.0)
	0.25	1.2 (0.6–1.8)	1.2 (0.6–1.8)	1.1 (0.6–1.7)	1.0 (0.5–1.6)	1.0 (0.5–1.5)		0.25	0.6 (0.2–1.1)	0.6 (0.2–1.1)	0.6 (0.2–1.1)	0.6 (0.2–1.0)	0.6 (0.2–1.0)
	0	1.0 (0.4–1.5)	1.0 (0.4–1.5)	1.0 (0.4–1.5)	0.9 (0.4–1.5)	0.9 (0.4–1.5)		0	0.6 (0.2–1.1)	0.6 (0.2–1.1)	0.6 (0.2–1.1)	0.6 (0.2–1.0)	0.6 (0.2–1.0)
2070							2070						
	1	0.8 (0.4–1.3)	0.8 (0.4–1.2)	0.7 (0.4–1.1)	0.6 (0.3–0.9)	0.5 (0.2–0.8)		1	0.5 (0.2–0.8)	0.5 (0.2–0.8)	0.5 (0.2–0.8)	0.5 (0.2–0.8)	0.5 (0.2–0.7)
	0.75	0.8 (0.4–1.2)	0.8 (0.4–1.1)	0.7 (0.4–1.0)	0.6 (0.3–0.9)	0.6 (0.3–0.8)		0.75	0.5 (0.2–0.8)	0.5 (0.2–0.8)	0.5 (0.2–0.8)	0.5 (0.2–0.8)	0.5 (0.2–0.7)
	0.5	0.7 (0.4–1.1)	0.7 (0.4–1.1)	0.7 (0.3–1.0)	0.6 (0.3–0.9)	0.6 (0.3–0.9)		0.5	0.5 (0.2–0.8)	0.5 (0.2–0.8)	0.5 (0.2–0.8)	0.5 (0.2–0.8)	0.5 (0.2–0.7)
	0.25	0.7 (0.3–1.0)	0.7 (0.3–1.0)	0.6 (0.3–0.9)	0.6 (0.3–0.9)	0.6 (0.3–0.9)		0.25	0.5 (0.2–0.8)	0.5 (0.2–0.8)	0.5 (0.2–0.8)	0.5 (0.2–0.7)	0.4 (0.2–0.7)
	0	0.6 (0.3–0.9)	0.6 (0.3–0.9)	0.6 (0.3–0.9)	0.6 (0.3–0.9)	0.6 (0.3–0.9)		0	0.5 (0.2–0.7)	0.5 (0.2–0.7)	0.5 (0.2–0.7)	0.4 (0.2–0.7)	0.4 (0.2–0.7)
2050							2050						
	1	0.4 (0.2–0.7)	0.4 (0.2–0.6)	0.4 (0.2–0.6)	0.3 (0.2–0.6)	0.3 (0.1–0.5)		1	0.4 (0.2–0.6)	0.4 (0.2–0.6)	0.4 (0.2–0.6)	0.4 (0.2–0.6)	0.3 (0.1–0.5)
	0.75	0.4 (0.2–0.6)	0.4 (0.2–0.6)	0.4 (0.2–0.6)	0.4 (0.2–0.6)	0.3 (0.1–0.6)		0.75	0.4 (0.1–0.6)	0.4 (0.1–0.6)	0.3 (0.1–0.6)	0.3 (0.1–0.6)	0.3 (0.1–0.5)
	0.5	0.4 (0.2–0.6)	0.4 (0.2–0.6)	0.4 (0.2–0.6)	0.4 (0.2–0.6)	0.3 (0.1–0.6)		0.5	0.3 (0.1–0.6)	0.3 (0.1–0.6)	0.3 (0.1–0.6)	0.3 (0.1–0.5)	0.3 (0.1–0.5)
	0.25	0.4 (0.2–0.6)	0.4 (0.2–0.6)	0.4 (0.2–0.6)	0.4 (0.2–0.6)	0.4 (0.2–0.6)		0.25	0.3 (0.1–0.5)	0.3 (0.1–0.5)	0.3 (0.1–0.5)	0.3 (0.1–0.5)	0.3 (0.1–0.5)
	0	0.4 (0.2–0.6)	0.4 (0.2–0.6)	0.4 (0.2–0.6)	0.4 (0.2–0.6)	0.4 (0.1–0.6)		0	0.3 (0.1–0.5)	0.3 (0.1–0.5)	0.3 (0.1–0.5)	0.3 (0.1–0.5)	0.3 (0.1–0.5)

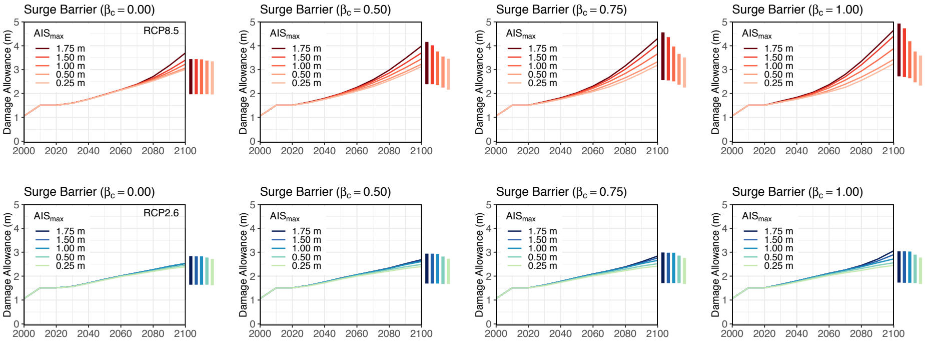

Figure B.7. **Top Row:** Instantaneous flood damage allowances (meters above mean higher high water [MHHW]) over time (2000–2100) for a storm surge barrier protecting Manhattan under different maximum 2100 Antarctic Ice Sheet (AIS) contribution thresholds (AIS_{max}, relative to 2000), different subjectively perceived likelihoods of AIS collapse (β_c; 0 being 'most unlikely' and 1 being 'most likely'), and for the representative concentration pathway (RCP) 8.5 climate forcing scenario. The colored bars in the margins of each plot show the 2100 damage allowances using only the 5/95th percentile local sea-level rise projections. The storm surge barrier allowances include 0.5 m of freeboard, have a 10% probability of failure at the design height, and the barrier gates close when water levels are > 1.0 m above MHHW. **Bottom Row:** As for Top Row, but for RCP2.6.

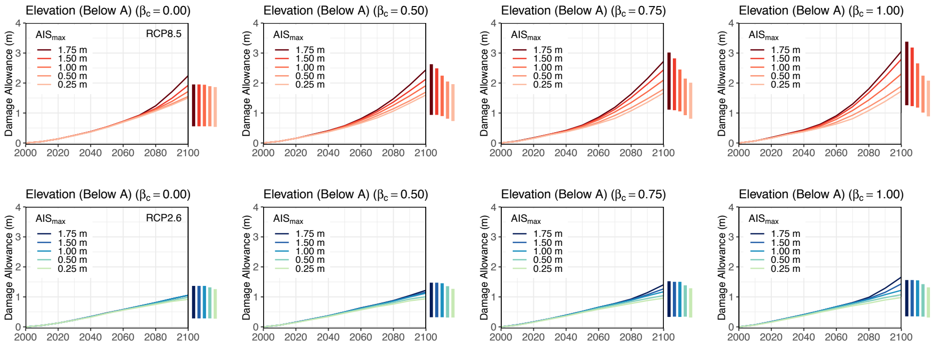

Figure B.8. **Top Row:** Instantaneous flood damage allowances (meters above mean higher high water [MHHW]) over time (2000–2100) for an elevation strategy below the damage allowance A for Manhattan under different maximum 2100 Antarctic Ice Sheet (AIS) contribution thresholds (AIS$_{max}$, relative to 2000), different subjectively perceived likelihoods of AIS collapse (β_c; 0 being 'most unlikely' and 1 being 'most likely'), and for the representative concentration pathway (RCP) 8.5 climate forcing scenario. The colored bars in the margins of each plot show the 2100 damage allowances using only the 5/95th percentile local sea-level rise projections. The damage allowances assume perfect compliance (i.e., all structures below A elevate). **Bottom Row:** As for Top Row, but for RCP2.6.

Table B.2. Levee damage allowances (meters above the current protection height) for 2100, 2070, and 2050 under representative concentration pathway (RCP) 8.5 and RCP2.6 and for different assumptions regarding future Antarctic ice sheet (AIS) behavior (e.g, likelihood of AIS collapse [β_c] and maximum 2100 AIS contribution [AIS_{max}]). Levee damage allowances include 0.5 m of freeboard and have a 10% probability of failure at the design height.

Levee Damage Allowances (m)

RCP8.5		AIS_{max}					RCP2.6		AIS_{max}				
	β_c	1.75 m	1.5 m	1.0 m	0.5 m	0.25 m		β_c	1.75 m	1.5 m	1.0 m	0.5 m	0.25 m
2100							2100						
	1.0	3.4	3.1	2.6	2.2	2.0		1.0	1.9	1.7	1.5	1.4	1.3
	0.75	3.1	2.8	2.4	2.1	2.0		0.75	1.7	1.6	1.5	1.4	1.3
	0.50	2.8	2.5	2.2	2.0	1.9		0.50	1.5	1.5	1.4	1.3	1.2
	0.25	2.6	2.2	2.1	1.9	1.9		0.25	1.4	1.4	1.4	1.3	1.2
	0.0	2.6	2.2	2.0	1.9	1.8		0.0	1.4	1.4	1.4	1.3	1.2
2070							2070						
	1.0	1.6	1.5	1.4	1.2	1.1		1.0	1.1	1.1	1.1	1.1	1.0
	0.75	1.5	1.4	1.3	1.2	1.1		0.75	1.1	1.1	1.1	1.0	1.0
	0.50	1.4	1.4	1.3	1.2	1.1		0.50	1.1	1.1	1.0	1.0	1.0
	0.25	1.3	1.3	1.2	1.2	1.2		0.25	1.0	1.0	1.0	1.0	1.0
	0.0	1.2	1.2	1.2	1.2	1.2		0.0	1.0	1.0	1.0	1.0	0.9
2050							2050						
	1.0	0.9	0.9	0.8	0.8	0.8		1.0	0.8	0.8	0.8	0.8	0.8
	0.75	0.9	0.9	0.8	0.8	0.8		0.75	0.8	0.8	0.8	0.8	0.8
	0.50	0.9	0.9	0.8	0.8	0.8		0.50	0.8	0.8	0.8	0.8	0.8
	0.25	0.8	0.8	0.8	0.8	0.8		0.25	0.8	0.8	0.8	0.8	0.7
	0.0	0.8	0.8	0.8	0.8	0.8		0.0	0.8	0.8	0.8	0.7	0.7

Table B.3. Coastal retreat damage allowances (meters above the current protection height) for 2100, 2070, and 2050 under representative concentration pathway (RCP) 8.5 and RCP2.6 and for different assumptions regarding future Antarctic ice sheet (AIS) behavior (e.g, likelihood of AIS collapse [β_c] and maximum 2100 AIS contribution [AIS_{max}]). Assumes perfect compliance of coastal retreat (i.e., $\alpha = 1$; Sec. 2.1.3).

Coastal Retreat Damage Allowances (m)

RCP8.5		AIS_{max}					RCP2.6		AIS_{max}				
	β_c	1.75 m	1.5 m	1.0 m	0.5 m	0.25 m		β_c	1.75 m	1.5 m	1.0 m	0.5 m	0.25 m
2100							2100						
	1.0	2.6	2.4	1.9	1.5	1.3		1.0	1.2	1.1	0.9	0.7	0.6
	0.75	2.3	2.0	1.7	1.4	1.3		0.75	1.0	0.9	0.8	0.7	0.6
	0.50	2.0	1.7	1.5	1.3	1.2		0.50	0.8	0.8	0.8	0.7	0.6
	0.25	1.8	1.5	1.4	1.2	1.2		0.25	0.8	0.8	0.7	0.6	0.6
	0.0	1.8	1.5	1.4	1.2	1.1		0.0	0.7	0.7	0.7	0.6	0.6
2070							2070						
	1.0	0.9	0.8	0.7	0.6	0.4		1.0	0.5	0.4	0.4	0.4	0.3
	0.75	0.8	0.7	0.7	0.5	0.5		0.75	0.4	0.4	0.4	0.4	0.3
	0.50	0.7	0.7	0.6	0.5	0.5		0.50	0.4	0.4	0.4	0.3	0.3
	0.25	0.6	0.6	0.6	0.5	0.5		0.25	0.4	0.4	0.4	0.3	0.3
	0.0	0.6	0.6	0.5	0.5	0.5		0.0	0.3	0.3	0.3	0.3	0.3
2050							2050						
	1.0	0.3	0.2	0.2	0.2	0.1		1.0	0.2	0.2	0.2	0.2	0.1
	0.75	0.2	0.2	0.2	0.2	0.2		0.75	0.2	0.2	0.2	0.1	0.1
	0.50	0.2	0.2	0.2	0.2	0.2		0.50	0.2	0.2	0.2	0.1	0.1
	0.25	0.2	0.2	0.2	0.2	0.2		0.25	0.1	0.1	0.1	0.1	0.1
	0.0	0.2	0.2	0.2	0.2	0.2		0.0	0.1	0.1	0.1	0.1	0.1

Table B.4. Damage allowances (meters) for a storm surge barrier for 2100, 2070, and 2050 under representative concentration pathway (RCP) 8.5 and RCP2.6 and for different assumptions regarding future Antarctic ice sheet (AIS) behavior (e.g, likelihood of AIS collapse [β_c] and maximum 2100 AIS contribution [AIS_{max}]). The allowances are relative to mean higher high water (MHHW). The storm surge barrier allowances include 0.5 m of freeboard, have a 10% probability of failure at the design height, and the barrier gates close when water levels are > 1.0 m above MHHW (approximately once every 10 years based on observations from the recent past; Fig. 1A).

Storm Surge Barrier Damage Allowances (m)

RCP8.5		AIS_{max}					RCP2.6		AIS_{max}				
	β_c	1.75 m	1.5 m	1.0 m	0.5 m	0.25 m		β_c	1.75 m	1.5 m	1.0 m	0.5 m	0.25 m
2100							2100						
	1.0	4.6	4.3	3.8	3.4	3.2		1.0	3.0	2.8	2.7	2.5	2.4
	0.75	4.2	4.0	3.6	3.3	3.1		0.75	2.8	2.7	2.6	2.5	2.4
	0.50	3.9	3.6	3.4	3.2	3.1		0.50	2.6	2.6	2.6	2.4	2.3
	0.25	3.7	3.4	3.3	3.1	3.0		0.25	2.5	2.5	2.5	2.4	2.3
	0.0	3.6	3.3	3.2	3.0	2.9		0.0	2.5	2.5	2.5	2.4	2.3
2070							2070						
	1.0	2.7	2.6	2.5	2.3	2.2		1.0	2.2	2.2	2.2	2.1	2.1
	0.75	2.6	2.5	2.4	2.3	2.2		0.75	2.2	2.2	2.2	2.1	2.1
	0.50	2.5	2.5	2.4	2.3	2.2		0.50	2.1	2.1	2.1	2.1	2.1
	0.25	2.4	2.4	2.3	2.3	2.2		0.25	2.1	2.1	2.1	2.1	2.0
	0.0	2.3	2.3	2.3	2.3	2.3		0.0	2.1	2.1	2.1	2.1	2.0
2050							2050						
	1.0	2.0	2.0	1.9	1.9	1.8		1.0	1.9	1.9	1.9	1.9	1.8
	0.75	2.0	1.9	1.9	1.9	1.9		0.75	1.9	1.9	1.9	1.8	1.8
	0.50	1.9	1.9	1.9	1.9	1.9		0.50	1.9	1.8	1.8	1.8	1.8
	0.25	1.9	1.9	1.9	1.9	1.9		0.25	1.8	1.8	1.8	1.8	1.8
	0.0	1.9	1.9	1.9	1.9	1.9		0.0	1.8	1.8	1.8	1.8	1.8

Table B.5. Damage allowances (meters) for an elevation strategy in which structures elevate below the allowance height (A; Fig. 2A) for 2100, 2070, and 2050 under representative concentration pathway (RCP) 8.5 and RCP2.6 and for different assumptions regarding future Antarctic ice sheet (AIS) behavior (e.g, likelihood of AIS collapse [β_c] and maximum 2100 AIS contribution [AIS_{max}]). The allowances are relative to the current protection height around Manhattan (assumed to be a bulkhead 1.0 m above mean higher high water [MHHW]). The elevation strategy assumes perfect compliance (i.e., all structures elevate).

Elevation (below A) Damage Allowances (m)

RCP8.5		AIS_{max}					RCP2.6		AIS_{max}				
	β_c	1.75 m	1.5 m	1.0 m	0.5 m	0.25 m		β_c	1.75 m	1.5 m	1.0 m	0.5 m	0.25 m
2100							2100						
	1.0	3.1	2.8	2.3	1.9	1.7		1.0	1.7	1.4	1.2	1.1	1.0
	0.75	2.7	2.4	2.1	1.8	1.7		0.75	1.4	1.3	1.2	1.0	1.0
	0.50	2.4	2.1	1.9	1.7	1.6		0.50	1.2	1.2	1.1	1.0	0.9
	0.25	2.3	1.9	1.8	1.6	1.6		0.25	1.1	1.1	1.1	1.0	0.9
	0.0	2.2	1.9	1.7	1.6	1.5		0.0	1.1	1.1	1.1	1.0	0.9
2070							2070						
	1.0	1.3	1.2	1.1	0.9	0.8		1.0	0.8	0.8	0.8	0.8	0.7
	0.75	1.2	1.1	1.0	0.9	0.8		0.75	0.8	0.8	0.8	0.7	0.7
	0.50	1.1	1.0	1.0	0.9	0.8		0.50	0.8	0.7	0.7	0.7	0.7
	0.25	1.0	1.0	0.9	0.9	0.9		0.25	0.7	0.7	0.7	0.7	0.7
	0.0	0.9	0.9	0.9	0.9	0.9		0.0	0.7	0.7	0.7	0.7	0.7
2050							2050						
	1.0	0.6	0.6	0.6	0.5	0.5		1.0	0.5	0.5	0.5	0.5	0.5
	0.75	0.6	0.6	0.6	0.5	0.5		0.75	0.5	0.5	0.5	0.5	0.5
	0.50	0.6	0.6	0.5	0.5	0.5		0.50	0.5	0.5	0.5	0.5	0.5
	0.25	0.6	0.5	0.5	0.5	0.5		0.25	0.5	0.5	0.5	0.5	0.5
	0.0	0.5	0.5	0.5	0.5	0.5		0.0	0.5	0.5	0.5	0.5	0.4

B.1 Flood damage allowances: multi-strategy approach

We present an example of how a multi-strategy approach could be designed using both coastal retreat and a levee for Manhattan (Fig. B.9A). For both 2070 and 2100, the flood damage risk (AAL) is mapped out for varying elevations below which coastal retreat occurs (A_1) and for the height of a levee (A_2, levee height is relative to A_1) assuming AIS_{max} = 1.50 m and $\beta_c = 0.0$ (Fig. B.9B). A user could select a preferred AAL and subsequently set the coastal retreat and levee heights. For example, to maintain the current AAL of $0.1 billion/yr in 2100 with coastal retreat below 2 m of elevation, a levee of roughly 0.75 m would need to be constructed. Additional heat maps could be used to depict alternative assumptions of future AIS behavior.

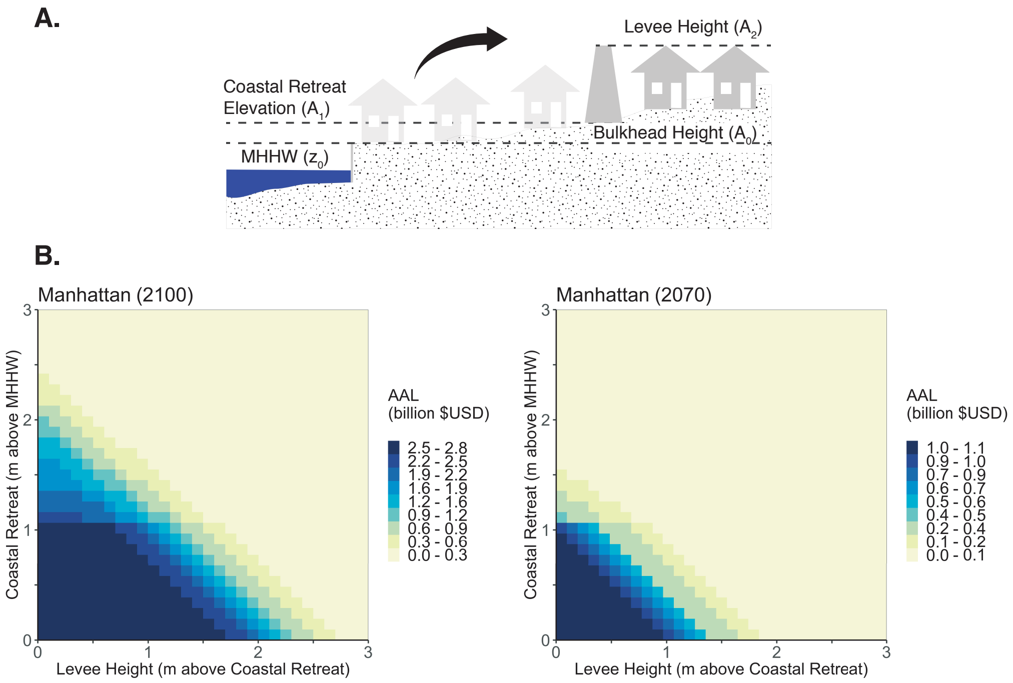

Figure B.9. **A.** Schematic depicting the combined implementation of coastal retreat and a levee. **B.** Heat maps of annual average loss due to flood damages for 2100 (Left) and 2070 (Right) using the flood protection strategies of coastal retreat (y-axis) and a levee (x-axis) with design heights between 0 and 3 m for each strategy. An additional 0.5 m of freeboard for the levee is not included in the depicted design heights along the x-axis. The levee has a 10% probability of structural failure at the design height.
